# Bio-inspired self-assembly of omega-3 fatty acids and peptides for responsive drug delivery

**DOI:** 10.1016/j.ijpx.2026.100558

**Published:** 2026-05-05

**Authors:** Simone Braccia, Luigi Alfano, Maria Carmen Ragosta, Rosa Bellavita, Gabriella D'Auria, Emanuela Esposito, Federica Donadio, Sara Palladino, Alessandro di Vaio, Rosa Camerlingo, Lucia Falcigno, Annarita Falanga, Michelino de Laurentiis, Antonio Giordano, Stefania Galdiero

**Affiliations:** a Department of Pharmacy, School of Medicine, University of Naples Federico II, Naples, Italy; b Sbarro Institute for Cancer Research and Molecular Medicine, Center for Biotechnology, College of Science and Technology, Temple University, Philadelphia, PA, USA; c Department of Breast and Thoracic Oncology, Istituto Nazionale Tumori – IRCCS – Fondazione G. Pascale, Naples, Italy; d Scuola Superiore Meridionale (SSM), Clinical and Translational Oncology Program, University of Naples Federico II, Naples, Italy; e Institute of Applied Sciences and Intelligent Systems (ISASI), Naples Cryo-Electron Microscopy Laboratory – EYE LAB, National Research Council (CNR), Naples, Italy; f Cell Biology and Biotherapy Unit, Istituto Nazionale Tumori-IRCCS-Fondazione G. Pascale, 80131 Naples, Italy; g Department of Agricultural Sciences, University of Naples Federico II, Portici, Italy; h SHRO Italia Foundation ETS, 10060 Candiolo, Turin, Italy; i Department of Medical Biotechnologies, University of Siena, 53100 Siena, Italy

**Keywords:** Drug delivery platform, Omega-3, Nanoparticles, Peptide, Cell penetrating peptide

## Abstract

Cancer treatments often yield unsatisfactory long-term clinical outcomes due to issues such as drug resistance, systemic toxicity, and suboptimal efficacy. Therefore, there is a need to develop novel drug delivery platforms for personalized medicine to enhance patient quality of life. In this study, an eicosapentaenoic acid (EPA)–based delivery system was developed and characterized. The system was functionalized with two different anticancer drugs, doxorubicin or pemetrexed, using an on-demand release strategy based on a proteolytic sequence specifically recognized by metalloproteinase-9 (MMP-9), an enzyme overexpressed in various cancers. We set-up the formulation procedure to obtain EPA-based nanoparticles (EPA-NPs) with a diameter of approximately 200 nm and low polydispersity, which were characterized for their aggregation properties and structural stability. The therapeutic efficacy was initially analyzed on HeLa cells as a cancer model due to their high transfection efficiency, reproducibility, and overexpression of MMP-9, using doxorubicin (Dox) as the drug. Enzyme-triggered drug release studies showed a rapid and controlled release profile, with approximately 80% of Dox released within 120 min in the presence of MMP-9. The efficient internalization was assessed using confocal microscopy; furthermore, Dox release induced an antiproliferative effect on HeLa cells (up to ∼60% cytotoxicity at 72 h) comparable to Dox free, while exhibiting no cytotoxicity toward healthy keratinocyte cells. Based on these results, the platform was later tested on a more disease-specific model, the mesothelioma, to confirm its relevance and adaptability for treating this aggressive cancer. These findings suggest that EPA nanoparticles could serve as a promising drug delivery platform.

## Graphical abstract

**Figure:**
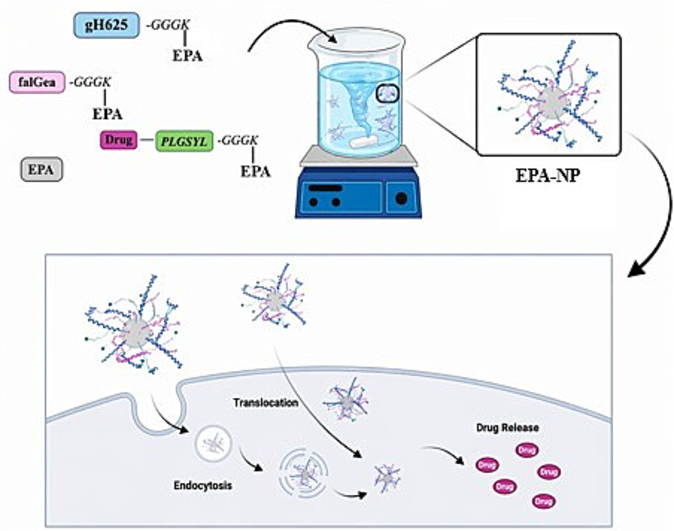

## 1 Introduction

Many chemotherapeutic agents have been developed, but their clinical success is frequently hindered by severe side effects and the non-specific uptake of drugs by healthy cells, which results in widespread toxicity and limits treatment effectiveness. In this context, nanomedicine and advanced drug delivery technologies offer a promising alternative, capable of improving therapeutic outcomes by enhancing efficacy, reducing systemic toxicity, prolonging circulation time, enabling targeted delivery to specific tissues, increasing patient compliance, and supporting the development of more innovative and effective treatment strategies (Bellavita et al., 2023; Del Genio et al., 2022a; Falanga et al., 2024). Over the past several decades, advances in drug delivery have enabled mRNA vaccines, which were vital during the COVID-19 pandemic (Park et al., 2021). Future efforts focus on improving tissue and cellular targeting; developing long-acting and controlled-release systems; enabling personalized delivery platforms; and integrating technologies such as artificial intelligence and machine learning to enhance precision and performance (Geng et al., 2025). The shift toward personalized medicine underscores the need for modular drug delivery systems that can be tailored to individual patient subtypes (Zheng et al., 2025; Liu et al., 2026). In this context, modular and biodegradable delivery platforms are essential to reduce toxicity and protect healthy tissues and organs (Waheed et al., 2024; Shameli et al., 2026). In fact, biodegradable delivery systems offer major advantages over non-biodegradable ones: they break down into non-toxic byproducts, reducing long-term accumulation; are less immunogenic due to their similarity to natural molecules; eliminate the need for surgical removal; enable controlled and sustained drug release; support localized and targeted therapy; and are environmentally friendly, degrading into harmless substances after use (Yun et al., 2025). Here; we developed a novel biodegradable platform for transport and release of drugs; using omega-3 fatty acids as the carrier. Omega-3 fatty acids have been previously reported in the literature as a therapeutic payload rather than as a structural component of the carrier system itself. Here; through a self-assembling process; these fatty acids form stable and modular nanoparticles that can be functionalized with targeting sequences; drugs; barrier penetrating moieties. In particular; fish-derived supplements; such as omega-3 (a group of polyunsaturated fatty acids); have gathered significant attention from researchers due to their numerous health benefits; including the prevention of inflammation; cancer; hyperlipidemia; diabetes; and cardiovascular diseases (Lobine et al., 2022; Tsugane, 2021). The three main types of omega-3 are alpha-linolenic acid (ALA), eicosapentaenoic acid (EPA), and docosahexaenoic acid (DHA).

Recent studies have highlighted the potential of EPA and DHA, as complementary options in cancer treatment (Fodil et al., 2022; Soni et al., 2022). Furthermore, when combined with doxorubicin (Dox), omega-3 has been shown to improve its benefits and lessen its negative side effects in a variety of cancers (Theinel et al., 2023; Pettersen et al., 2023). Early clinical trials suggest that breast cancer patients undergoing anthracycline therapy may suffer less cardiac damage when treated with carvedilol, a β-blocker with antioxidant effects, alongside DHA, which boosts the heart's natural antioxidant defenses (Carrasco et al., 2020). Several recent studies have also explored the combined use of EPA/DHA and Dox in cancer treatment (Jameel et al., 2024; Crovella et al., 2023; Gurav et al., 2023).

Thus, omega-3 represents a promising class of self-assembling skeletons that have great potential for generating new cancer therapeutics (Fattahi et al., 2020; Li et al., 2022). Prodrugs that may self-assemble or be stably entrapped in various delivery matrices may be created by covalently conjugating omega-3 to hydrophobic or hydrophilic drug molecules *via* a cleavable linkage (Huang et al., 2021; Wu et al., 2020). Some initial attempts to incorporate omega-3 into drug delivery systems have been reported, though none aim to develop a platform composed entirely of omega-3. For instance, DHA has been shown to inhibit tumor growth and enhance the antitumor efficacy of PTX and docetaxel (Wang et al., 2021a); leading to its incorporation into polymer-based nanoparticles for cancer treatment (Wang et al., 2021a; Lanna et al., 2021; Xie et al., 2020; Chen et al., 2022; Wang et al., 2021b).

Herein, we describe the first development of a nanoplatform for drug delivery made completely of EPA, which enables the formation of a biodegradable nano delivery system that once degraded will release EPA, able to further perform its beneficial effect on the target site. Our EPA-based nanoparticles (EPA-NPs) offer several advantages over other polymeric nanoparticles; in fact, they are derived from natural sources and are biocompatible, reducing the risk of adverse reactions or toxicity. Furthermore, EPA-based nanoplatforms possess numerous health properties, providing additional therapeutic benefits beyond drug delivery. Our EPA-NPs can be modified to include surface modifications that enhance their specificity to target cells or tissues and improve drug delivery efficiency, and they do not have environmental implications during production and disposal. Compared to lipid-based delivery systems, including lipid nanoparticles, fatty-acid-based carriers, and self-assembled prodrug platforms, which generally rely on multicomponent formulations or chemical conjugation to form stable nanoparticles or to the use of omega-3 fatty acids as auxiliary or bioactive components, the platform presented here consists entirely of unconjugated EPA, as the sole structural material, enabling spontaneous self-assembly in an excipient-free, single-component system.

As proof of concept, we engineered EPA-NPs decorating their surface with 1) a cell penetrating peptide gH625, developed in our laboratory, which enhances penetration across cell membranes (Bellavita et al., 2024; Del Genio et al., 2022b; Falanga et al., 2018; Guarnieri et al., 2015; Guarnieri et al., 2013; Valiante et al., 2015); 2) a drug (Doxorubicin (Dox) or Pemetrexed (PEM)), linked to the nanosystem through a peptide cleavable sequence recognized by the matrix metalloproteinase-9 (MMP-9) enabling site-specific, on-demand release triggered by the enzyme's overexpression in the tumor microenvironment (Bellavita et al., 2024); and 3) a tumor-targeting peptide sequence. The novelty of our platform lies in the integrated and modular combination of an EPA-based matrix used as a structural carrier (rather than a payload), a multifunctional peptide enabling targeting, enzymatic responsiveness, and enhanced internalization, which together provide a coordinated and biocompatible system that, while building on existing components, represents a meaningful advancement in multifunctional biocompatible nanocarrier design.

We set up the formulation procedure to obtain EPA-NPs with suitable dimensions for therapeutic applications. EPA-NPs with different compositions in terms of EPA and EPA-based peptides were prepared and analyzed for their aggregation properties, dimensions, morphology and structural stability exploiting dynamic light scattering, circular dichroism, ^1^H NMR, scanning electron microscopy. To confirm the cellular and nuclear internalization of EPA-NPs, we used confocal microscopy.

Initial biological experiments were performed on HeLa cells, which were chosen as a model system due to their well-established use in biomedical research and their high transfection efficiency. HeLa cells are known to overexpress MMP-9, making them an ideal *in vitro* model for evaluating both the internalization efficiency of our EPA-based delivery platform and the enzyme-triggered drug release mechanism.We used the drug Dox, which allowed us to follow the drug location in the cell after release from EPA-NPs. The robustness, reproducibility, and relevance of this model to cancer biology provided a reliable foundation for assessing the cellular uptake, localization, and therapeutic efficacy of our EPA-NPs before transitioning to more disease-specific models. Building on these findings, we later transitioned to mesothelioma cancer models - malignant MSTO-211H cells and non-malignant MET-5 A cells- to assess the platform performance in clinically relevant and disease-specific context, given the aggressive nature of mesothelioma and its limited treatment options. This step allowed us to demonstrate the adaptability and potential translational relevance of our delivery system.

## 2 Materialsandmethods

### 2.1 Materials

Eicosapentaenoic Acid (EPA) was purchased from Carbosynth (Berkshire, UK) with a melting point of −54 °C. Fmoc-Ala-OH, Fmoc-Arg(Pbf)-OH, Fmoc-Gly, Fmoc-His(Trt)-OH, Fmoc-Leu-OH, Fmoc-Ser(tBu)-OH, Fmoc-Thr(OtBu)-OH, Fmoc-Trp(Boc)-OH, Fmoc-Tyr(tBu)-OH, Fmoc-Asn(Trt)-OH, Fmoc-Ile-OH, Fmoc-Phe-OH, Fmoc-Glu(OtBu)-OH, were acquired from GL Biochem Ltd. (Shanghai, China). Rink amide *p*-methylbenzhydrylamine (MBHA) resin, Fmoc-D-Phe-OH, Fmoc-D-Ala-OH, Fmoc-D-Leu-OH, Fmoc-D-Glu(OtBu)-OH, N,N′-diisopropylcarbodiimide (DIC), Oxyma pure, 1-[Bis(dimethylamino)methylene]-1H-1,2,3-triazolo[4,5-*b*]pyridinium 3-oxid hexafluorophosphate (HATU), *N,N*-Diisopropylethylamine, triisopropylsilane (TIS), 2-propanol, ethanol, 1-Hydroxybenzotriazole hydrate (HOBt), *N,N,N′,N′*-fbTetramethyl-O-(1H-benzotriazol-1-yl)uronium hexafluorophosphate (HBTU), Nile Red, Aldoxorubicin, Rhodamine B, Tetrakis(triphenylphosphine)palladium(0) Pd(PPh_3_)_4_, 1,3-Dimethylbarbituric acid (NDMBA), Nile Red, Aldoxorubicin, Rhodamine B, and 1,1,1,3,3,3-Hexafluoro-2-propanol (HFIP) were purchased from Merck (Italy). Fmoc-L-Glu(OAll)-OH, Fmoc-L-Lys(Mtt)-OH, piperidine, and trifluoroacetic acid (TFA) were bought from Iris Biotech GmbH (Marktredwitz, Germany). Anhydrous solvents [*N,N*-dimethylformamide (DMF) and dichloromethane (DCM)], were bought from Merck (Milan, Italy). Pemetrexed was purchased from MedChemExpress. All chemicals and reagents were of analytical grade and used as received.

### 2.2 Peptide synthesis and purification

Rink Amide resin (0.63 mmol/g as loading) was used as a solid support for the peptide synthesis. After two minutes of treatment with a 30% piperidine solution in DMF, the Fmoc protective group was eliminated. Fmoc-Lys(Mtt)-OH was used as the first amino acid for each peptide to perform the conjugation of EPA on the amine group in the lysine side chain (Fig. S1). Two coupling steps were used for each reaction coupling. In the first one, Fmoc-amino acid (2 equiv) was added with *N,N′-*diisopropylcarbodiimide (DIC, 2 equiv) oxyma pure (2 equiv) as coupling reagents, in DMF for 40 min; in the second one, Fmoc-amino acid (2 equiv) was added with HATU (2 equiv), DIPEA (4 equiv), in DMF for 40 min. To covalently conjugate the fatty acid, the lysine's Mtt group on the side chain was removed. The resin was treated with a DCM:TFA:TIS (94:1:5, *v*/v/v) cocktail to accomplish the Mtt deprotection. The process was carried out fifteen times at room temperature for 20 min per cycle. The progress of the deprotection was monitored using the Kaiser test**,** which is used to detect primary amines in the solid phase, and was utilized to determine the full Mtt removal. Then, the fatty acid conjugation was performed using Eicosapentaenoic acid (2 equiv), DIC (2 equiv), oxyma pure (2 equiv) in NMP overnight, and the second coupling was performed using HATU (2 equiv) and DIPEA (4 equiv), in NMP for 2 h. The labeled peptide was obtained performing the coupling with Rhodamine B (Rho) (2 equiv), HoBt (2equiv), HBTU (2equiv), DIPEA (4equiv) as coupling reagent, in DMF for 3 h. The peptides were cleaved from the resin with a solution of TFA/H2O/TIS (95/2.5/2.5, *v/v/v*) under agitation for 3 h. Then, peptides were precipitated in diethyl ether and separated by centrifugation (2 × 15 min, 6000 rpm). The peptides were dissolved in HFIP (20%) and H_2_O (0.1% TFA) and purified by RP-HPLC semi-preparative (Jasco) on a Phenomenex Kinetex C18 column (5 μm, 100 Å, 150 × 21.2 mm) column, with a linear gradient of solvent B (0.1% TFA in acetonitrile) in solvent A (0.1% TFA in water) from 10 to 90% in 20 min with flow rate of 15 mL/min and UV detection at 220 nm. Then, the pure peptides were analyzed by the analytical HPLC (Jasco LC-NetII/ADC) through a Phenomenex Jupiter 4u Proteo column, 90 Å, 150 × 4.6 mm with a linear gradient from 10 to 90% in 15 min (Fig. S2-S8). All peptides involved in EPA-NPs formation are listed in Table 1.

**Table 1:** Peptide sequences used in EPA-based nanoparticle formation.

Peptide	Sequence
gH-EPA	NH₂-HGLASTLTRWAHYNALIRAF-GGG-K(EPA)-CONH_2_
Rho-gH-EPA	Rho-HGLASTLTRWAHYNALIRAF-GGG-K(EPA)-CONH₂
falGea-EPA	NH₂-falGea-GGG-K(EPA)-CONH₂
Dox-EPA-NC	Ac-C(Dox)-GGG-K(EPA)-CONH₂
Dox-EPA	Ac-C(Dox)-PLGSYL-GGG-K(EPA)-CONH₂
PEM-EPA	Ac-E(PEM)-PLGSYL-GGG-K(EPA)-CONH₂

### 2.3 Doxorubicin conjugation

Doxorubicin was covalently linked to the free thiol group of a cysteine residue introduced in the *N*-terminus of the peptide Ac-Cys-PLGSYL-GGG-K(EPA). The peptide Ac-Cys-PLGSYLGGG-K(EPA) with the acetyl group in *N*-terminus was synthesized and purified as described above. The conjugation was performed between the maleimide moiety of aldoxorubicin (2 equiv) and cysteine (2 equiv) in solution (Xie et al., 2020). The reaction was performed dissolving the peptide in DMF at a concentration of 1 mg/mL and aldoxorubicin at 1 mg/mL. The peptide solution was stirred on a magnetic stirring plate at 40 °C, while aldoxorubicin was added dropwise using a syringe pump at a flow rate of 25 μL/min. Additionally, DIPEA (5 eq) was added to the mixture. After the complete addition of the drug to the peptide, the reaction was stirred for 1 h at 40 °C and then allowed to proceed overnight at room temperature. DMF was subsequently removed, and the compound was lyophilized. The obtained compound was purified by preparative HPLC. The reaction was monitored by NMR. (Supplementary material Fig. S9 Panel A).

### 2.4 Pemetrexed conjugation

To enable pemetrexed conjugation, a peptide incorporating the MMP-9 cleavable sequence Pro-Leu-Gly-Ser-Tyr-Leu was synthesized. This sequence was separated from a C-terminal lysine, used for EPA attachment, by a tri-glycine linker. An N-terminal glutamic acid residue protected with allyl group was also included, acetylated at its α-amino group, and pemetrexed was coupled through the γ-carboxyl group of its side chain after its deprotection. In particular, the allyl group was removed using a solution of Pd(PPh_3_)_4_ (0.15 equiv) and NDMBA (3 equiv) in dry DCM/DMF (3:2, *v*/v), and gently shaken for 1 h under Ar. After filtering, the resin was washed with DMF and DCM and allowed to dry. The process of allyl deprotection was then carried out a second time. After washing the resin with a 0.5% sodium *N,N*-diethyldithiocarbamate solution in DMF (30 min × 2), LC − MS analysis was used to confirm that all the allyl groups had been removed. Prior to coupling, both carboxylic acid groups of pemetrexed were protected in solution by converting the drug into its dimethyl ester derivative PEM(OMe)₂ using SOCl₂ in methanol, following the procedure described by Miklán et al (Miklan et al., 2011). Then, the protected drug (3 equiv) was then conjugated to the carboxylic group of glutamic residue using PyAOP (3 equiv), DIPEA (6 equiv), and PEM(OMe)₂ (3 equiv) as coupling reagents, in DMF as solvent. After the reaction ascertained by LC-MS analysis, the peptide was removed from the resin, while the methyl ester protecting groups were removed by dissolving the crude in a 1:1 (*v*/v) mixture of 0.1 M NaOH and acetone for 60 min. Sodium hydroxide was used in approximately 2.5-fold molar excess relative to the methyl esters. The deprotection process was monitored by RP-HPLC. The solution was subsequently neutralized by the addition of 200 μL of 0.1 M HCl. Finally, the peptide was purified by HPLC, and the identity and structural integrity were confirmed by nuclear magnetic resonance (NMR) spectroscopy. (Supplementary material Fig. S9 Panel B).

### 2.5 NMR Experimental section

NMR analyses of Ac-C(Dox)-GGG-K(EPA)-CONH₂ and Ac-C(Dox)-PLGSYL-GGG-K(EPA)-CONH₂ were carried out in the organic solvent CD₃OD (99.8% D, Sigma Aldrich) for their low solubility at 298 K using a 700 MHz Bruker Neo Avance spectrometer situated at the Department of Pharmacy – University “Federico II” of Naples and furnished with a z-gradient 5 mm triple-resonance cryoprobe. Sample solutions were prepared by dissolving weighted amounts of products in 600 μL of CD₃OD. The solutions appeared clear and reddish. 1D proton NMR spectra were recorded with an interscan delay (d₁) time of 10 s and analyzed using MESTRENOVA 6.0 software (Mestrelab Research, S.L., Santiago de Compostela, Spain). 2D homonuclear spectra such as TOCSY (mixing time 70 ms) and NOESY (mixing time 300 ms) were recorded in the phase-sensitive mode using the States method, with 4096 data points in t₂ and 512 equidistant t₁ values. Heteronuclear (^1^H—^13^C) HSQC spectra were acquired using 2048 data points in t₂ and 512 equidistant t₁ values. The residual water signal was suppressed using gradients. All 2D spectra were analyzed using the CARA program. Chemical shifts were referenced to the residual CHD₂ proton of methanol: 3.35 ppm for ^1^H and 49.3 ppm for ^13^C.

Similarly, NMR analysis of PEM-EPA (Ac-E(*PEM*)PLGSYL-GGG-K(EPA)-CONH₂) was carried out in DMSO‑*d*₆ (99.8% D, Sigma Aldrich) for its low solubility at 298 K using the same instrument and conditions. Sample solution was prepared by dissolving weighted amounts of product in 600 μL of DMSO‑*d*₆. 1D proton NMR spectrum was recorded with an interscan delay (d₁) of 10 s and analyzed using MESTRENOVA 6.0 software. 2D homonuclear spectra, including TOCSY (mixing times of 30 and 70 ms) and NOESY (mixing time 300 ms), were acquired in the phase-sensitive mode using the States method, with 4096 data points in t₂ and 512 equidistant t₁ values. Heteronuclear (^1^H—^13^C) HSQC spectra were similarly acquired with 2048 data points in t₂ and 512 in t₁. Residual water signals were suppressed using gradients. All 2D spectra were analyzed using the CARA program. Chemical shifts were referenced to residual CHD₂ protons of DMSO: 2.49 ppm for ^1^H and 39.7 ppm for ^13^C.

### 2.6 Preparation of EPA nanoparticles

EPA nanoparticles were prepared by the nanoemulsion-solvent evaporation methodology based on low energy. Ethanol (EtOH), a water-miscible solvent, was used to dissolve the EPA based peptides at varying ratios. The resulting solution was added dropwise to the aqueous phase using a syringe pump (flow rate 50 μl/min) with a 20-gauge needle, to water phase under magnetic stirring at 1500 rev/min. The EtOH to water ratio was 1:2. Then, the organic solvent was removed using a rotary evaporator to obtain EPA-NPs in only water.

### 2.7 Size and zeta potential measurements

Dynamic light scattering (DLS) measurements were performed using a Zetasizer Nano-ZS (Malvern Instruments, Worcestershire, UK) to determine the dimensions and the zeta potential of the different EPA-NPs preparations. Measurements were performed in triplicate with He—Ne laser 4 mW operating at 633 nm at scattering angle fixed at 173° and at 25 °C, and each run consisting of 10 cycles. A 30-s delay was set between consecutive measurements.

### 2.8 Reverse titration to determine Critical Aggregation Concentration (CAC)

CAC of EPA alone and EPA conjugated peptides were determined by a fluorescence assay using Nile Red, a solvatochromic probe. Nile Red tends to locate itself into hydrophobic domains such as aggregated systems, inducing a blue shift and hyperchromic effect. A stock of EPA at 3000 mM in ethanol was added to an aqueous solution of Nile Red at 500 nM to carry out a titration, by adding a small volume of ethanol solution (approximately 0.5–1 μl), avoiding the blue shift given by the presence of huge ethanol volume. After each addition, the cuvette was shaken, and the measurement was subsequently performed in the range of 0.5–30 mM of EPA and EPA conjugated peptides. The emission spectra of Nile Red for each addition were measured by a Cary Eclipse Varian spectrometer. For every sample, at least three measurements of the Nile Red emission spectra (excitation wavelength 550 nm, emission wavelength range 570 to 700 nm) were made. The data obtained from three independent measurements (*n* = 3) were analyzed by plotting the maximum emission fluorescence corresponding wavelength (y) as a function of peptide concentration (x) and fitting with the sigmoidal Boltzmann equation (OriginPro Program for graphs):

$$y=\frac{A1-A2}{1+e^{\left(x-x0\right)/\mathrm{\Delta x})}}+A2$$

In the equation, A1 and A2 are two variables corresponding to the upper and lower limits of the sigmoid, respectively. Whereas x0 and Δx indicate the inflection point and the steepness of the sigmoid, respectively.

### 2.9 Drug loading

Dox and PEM were loaded on EPA-NPs surface driven by hydrophobic interactions of EPA. The drug loading (DLC) was experimentally calculated according to the following equation:

$$\mathrm{DLC}\;\left(\%\right)=\frac{\mathrm{weight of drug in the nanoparticle}}{\mathrm{weight of}\;\mathrm{EPA}-\mathrm{NPs}}\times 100$$

DLC can be directly read out from calibration lines, Supplementary material Fig. S10.

### 2.10 Morphological characterization by Scanning Electron Microscopy (SEM)

Nanoparticles EPA-gH, EPA-gH-Dox and EPA-gH-TP-PEM were prepared at the concentration of 200 μM and analyzed by SEM analysis. 5 μL drops of each formulation were spotted on a clean silicon wafer and air-dried at room temperature. The image was obtained by using dual beam FIB- SEM Aquilos 2 by ThermoFisher Scientific. The acquisition parameters were Current 0.2 nA, Voltage 7.5 kV, Working Distance 2.6 mm, Field of View 4.14 μm, Stage Tilt 0 and Magnification 50,000×.

### 2.11 Circular Dichroism (CD) analysis

The cell penetrating peptide bound to EPA (gH-EPA) for CD studies were prepared in Phosphate Buffer 5 mM and in presence of 20% 2,2,2-trifluoroethanol (TFE) at a final concentration of 8 μM. CD spectra were recorded from 200 nm to 260 nm using a Jasco J-180 spectropolarimeter with a 1.0 cm quartz cell at room temperature under nitrogen flow. Each spectrum was recorded three times, and we converted into mean molar ellipticity.

### 2.12 *In vitro* Doxo release by proteolytic cut of MMP-9

The formulation EPA-gH-Dox was prepared at the concentration of 350 μM. The enzyme was pre-activated with APMA 100 μM and Tris-HCl 50 mM (pH 7.2) and left at 37 °C for 3 h. The formulation was prepared at 350 μM and diluted at 175 μM with buffer solution: 50 mM HEPES, 200 mM NaCl, 10 mM CaCl, and 1 mM ZnCl_2_, at pH 7. Then, nanoparticles were incubated with MMP-9 (40 nM) at 37 °C. At each time point (30, 60, and 120 min), 50 μL was taken from the mixture, centrifuged at 13.000 rpm for 30 min and the supernatant was analyzed by UV/vis spectroscopy (NanoDrop ™ 2000/2000C, Jasco, Milan, Italy) following absorbance at 480 nm (Dox).

### 2.13 Serum stability of EPA-gH-Dox

The serum stability of EPA-gH-Dox was evaluated by DLS. EPA-gH-Dox was prepared at a final concentration of 350 μM and incubated with 10% of fetal bovine serum (FBS) at 37 °C for different times (0, 3, 24, 48, 72 h). At increasing time intervals, an aliquot of nanoparticles was taken and centrifuged at 14.000 ×*g* rpm for 30 min at room temperature in the presence of 90% ACN. The supernatant was analyzed by DLS, and the stability of PA-gH-Dox was evaluated following the size intensity of the nanoparticles. The experiment was conducted in triplicate.

### 2.14 Cell cultures

HeLa, MSTO-211H and MET-5 A cell lines were obtained from the American Type Culture Collection _2_(ATCC, CCL-2) and cultured in Roswell Park Memorial Institute (RPMI) 1640 medium (Thermo Fisher Scientific). The medium was supplemented with 10% fetal bovine serum (FBS, Thermo Fisher Scientific), penicillin (100 U/ml), streptomycin (100 μg/ml), and 2 mM glutamine. Cells were maintained at 37 °C in a humidified atmosphere containing 5% CO₂. The MSTO-211H cell line (biphasic mesothelioma) and MET-5 A cell line (normal mesothelial epithelial cells) were cultured under the same conditions. Human HaCaT keratinocyte cell line was cultured in DMEM (Sigma) supplemented with 10% fetal bovine serum (FBS, Cambrex, Verviers, Belgium), l-glutamine (2 mM, Sigma), penicillin (100 units/ml, Sigma), and streptomycin (100 μg/ml, Sigma), and maintained in a humidified 5% CO₂ atmosphere at 37 °C, according to the supplier's recommendations.

### 2.15 Fluorescence confocal spectroscopy

The uptake of EPA-NPs by HeLa and MSTO-211H cells *in vitro* was studied by confocal microscopy following the Rhodamine B fluorescent signal bound to peptide gH-EPA. After 1 h incubation, cells were washed twice with PBS and fixed with 4% paraformaldehyde for 20 min. For HeLa cells, the final concentration of the nanosystem was 175 μM, containing gH-EPA in a final concentration of 10 μM. For MSTO-211H cells, the final concentration of the EPA-gH-TP-PEM system was 100 μM, with 6% of gH-EPA peptide labeled with Rhodamine B. Nuclei were stained using Hoechst. The immunofluorescence assay was acquired using the Zeiss confocal microscope LSM900 Airyscan 2, and image analysis was performed with Zen software.

The inhibition of active metabolism pathways was evaluated in HeLa cells by treatment with 40 μM sodium azide (NaN₃), added to the culture medium for 30 min (pre-treatment). Subsequently, the same NaN₃ concentration was maintained for the entire duration of the sample incubation for 3 h. The samples tested included: (i) EPA-gH-Dox system (350 μM), monitored by intrinsic doxorubicin fluorescence; (ii) EPA-gH (350 μM), assessed by the gH-Rho-labeled peptide signal. All formulations were diluted 1:1 with the control medium. For each preparation, two experimental conditions were set up: cells pre-treated with NaN₃ (40 μM) for 30 min, with subsequent removal of the medium and incubation with the samples for 3 h in the presence of NaN₃ (40 μM); cells incubated with the samples in the absence of NaN₃, as a control.

### 2.16 Antibodies and Western Blotting

The following primary antibodies were employed: MMP9 (N-terminal) polyclonal antibody (1:1000, #10375–2-AP, Proteintech), anti-EGF Receptor (D38B1) XP® Rabbit mAb (1:1000, #4267, Cell Signaling Technology), beta-actin antibody (1:1000, #sc-47,778, Santa Cruz Biotechnology) and the secondary antibodies mouse anti-rabbit IgG-HRP (1:5000, #sc-2357, Santa Cruz Biotechnology), m-IgGκ BP-HRP (1:5000, #sc-516,102, Santa Cruz Biotechnology). Total protein extraction was performed by lysing cells on ice at 4 °C using a buffer containing 50 mM HEPES (pH 7.5), 1% Triton X-100, 150 mM NaCl, and 5 mM EGTA, supplemented with a cocktail of protease and phosphatase inhibitors (Roche Applied Science, Penzberg, Germany). Cell lysates were clarified by centrifugation at 13,000 ×*g* for 20 min at 4 °C*. prior* to the addition of the primary antibody, membranes were blocked with 5% milk in 1× TBS-T (Tris-buffered saline with 0.1% Tween-20) for 1 h at room temperature. The primary antibodies were incubated with gentle agitation overnight at 4 °C, followed by three 10-min washes in 1× TBS-T. The secondary antibody was incubated for 1 h at room temperature, followed by another series of three 10 min washes in 1× TBS-T. For visualization, the nitrocellulose membrane was incubated for 5 min with Thermo Scientific Pierce ECL Western Blotting Substrate. Chemiluminescent signals were captured using the Invitrogen iBright Imaging Systems 1500. Western blot analysis was conducted on cell lysates using the mentioned antibodies, with beta-actin serving as the protein loading control.

### 2.17 γ-H2AX histone phosphorylation-immunofluorescence analysis

HeLa cells were cultured on glass coverslips and fixed using 4% paraformaldehyde. After fixation, the cells were permeabilized with 0.2% Triton X-100. Blocking was performed at room temperature (RT) for 10 min with 1% BSA. Cells were subsequently incubated for 1 h at 37 °C with the following primary antibodies: γ-H2AX S-139 (1:200, ab2893, Abcam). Following washes, cells were incubated for 45 min at 37 °C with secondary antibody: AlexaFluor 647 donkey anti-rabbit IgG (H + L) from ThermoFisher Scientific. Finally, cells were mounted onto glass slides using ProLong™ Gold Antifade Mountant with hoechst (ThermoFisher Scientific). Images were acquired using a Zeiss LSM 900 AiryScan2 microscope equipped with a 63×/1.4 oil objective, and analysis was performed with Zen software.

### 2.18 Cell viability assay

HeLa cells were seeded in triplicate into 96-well plates at a density of 1500 cells per well and allowed to adhere for 24 h. Subsequently, cells were treated with the indicated concentrations of the drug and incubated for an additional 48 or 72 h. At the end of the treatment period, cells were fixed with 50% *v*/v trichloroacetic acid and stained with 0.4% *w*/*v* sulpho-rhodamine B (SRB) in 1% v/v acetic acid. Cell viability was quantified relative to untreated controls, which were defined as 100%.

MSTO-211H and MET-5 A were seeded in triplicate in 96-well plates at a density of 3000 cells per well and treated with the indicated drug concentrations. After 48 and 72 h of incubation, cell viability was assessed following the same colorimetric assay based on SRB staining, as described for HeLa cells.

### 2.19 Bioscreens *in vitro*

The biocompatibility of nanosystems was studied by estimating a “cell survival index” resulting from combining cell viability assessment with cell counting (Ferraro et al., 2021). The cell survival index is the arithmetic mean between the percentage values derived from the MTT assay and automated cell counting. HaCaT cells were inoculated into 96-microwell culture plates at a density of 10^4^ cells/well and allowed to grow for 24 h. The medium was then replaced with fresh medium, and the cells were treated for an additional 48 and 72 h with Dox (10 and 20 μM), EPA-gH-Dox (150 and 300 μM), EPA (150 and 300 μM), EPA-gH (150 and 300 μM). The viability was assessed by the MTT assay (Sigma), which is based on the redox ability of living mitochondria to convert dissolved MTT into insoluble purple formazan. Briefly, after treatments, the medium was removed, and cells were incubated with 20 μL/well of MTT solution (5 mg/mL) for 1 h in a humidified 5% CO_2_ incubator at 37 °C. The incubation was stopped by removing the MTT solution and adding 100 μL/well of DMSO to solubilize the resulting formazan. Finally, absorbance was monitored at 550 nm using a microplate reader (ThermoFisher). Cell count was determined by the TC20 automated cell counter (Bio-Rad), which provides an accurate and reproducible total cell count and live/dead ratio in a single step using a specific dye (Trypan Blue) exclusion assay. Operationally, after treatments in 96-well culture plates, the medium was removed, and cells were harvested. Ten microliters of cell suspension, mixed with 0.4% Trypan Blue solution in a 1:1 ratio, were loaded into disposable slide chambers. Results are expressed as total cell count (number of cells per mL). If trypan blue is detected, the instrument also takes the dilution into account and displays the live cell count and percentage viability. Concentration effect curves were obtained with nonlinear regression using GraphPad Prism 8.0 curve-fitting program and are expressed as the mean values ± SEM (*n* = 30).

### 2.20 Citofluorimetric analysis

**Fig. 1:**
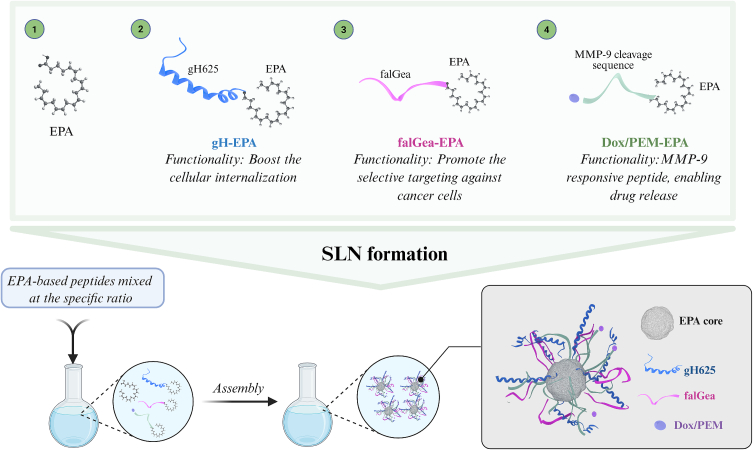
Schematic illustration of the peptides used in this study and the assembly of EPA-NPs, driven by hydrophobic interactions of EPA, within each moiety, which promote the exposure of peptide-based moieties on the surface. The figure was created with BioRender.

HeLa cells were treated as described in each Fig. 1 × 10^6^ cells were fixed for 20 min at room temperature with 4% paraformaldehyde and stored until cytofluorimetric analysis. Before FACS analysis, the cells were washed three times with 1× PBS.

### 2.21 Statistical analysis

Statistical analysis was performed using GraphPad Prism software (version 9 for Mac). Differences between two groups were evaluated using the Student's *t*-test, and *p* < 0.05 was considered statistically significant. The number of independent replicates and *p*-values are reported in the figure legends. Differences between more than two groups were evaluated using the ANOVA statistical post-test.

## 3 Results and discussion

### 3.1 EPA-based nanoparticles design and synthesis of peptide sequences for nanoparticle formation

We designed and produced structurally defined EPA-NPs using a mixture of EPA and EPA-based peptides (Table 1) bearing different biological moieties that were meant to be on the surface of the nanoparticles. Thus, EPA supplies the hydrophobic driving force for the nanoparticle assembly allowing the peptide-based moieties to be exposed on the surface.

In our assembly design, the nanoparticle surface was decorated with distinct peptide motifs to optimize cellular uptake and enhance the functionality of EPA-NPs (Fig. 1). The formulation of our EPA-NPs was guided by a rational design approach aimed at achieving precise self-assembly, optimal drug loading, and targeted delivery. The EPA amphiphilic properties ensured incorporation into the nanoparticle through the self-aggregation process, but the ratios of each motif covalently bound on the surface of EPA-NPs were systematically optimized to balance nanoparticle stability, size, and surface properties, while maintaining the therapeutic payload as reported in the following paragraphs. Overall, each step, from the selection of EPA to the covalent conjugation of the drug–peptide and the optimization of component ratios, was carefully designed to integrate structural stability with controlled, site-specific therapeutic activity.

To efficiently deliver EPA-NPs across membrane cellular barriers, suitable carriers must be selected. To this aim, we conjugated the cell-penetrating peptide gH625 (sequence: HGLASTLTRWAHYNALIRAF) to the EPA using a solid-phase approach. The peptide gH625, developed and extensively studied by our research group, is known to enhance cell membrane penetration and cross the blood-brain barrier by inducing temporary lipid membrane rearrangements, making it valuable for drug delivery applications (Del Genio et al., 2022a; Bellavita et al., 2024; Del Genio et al., 2022b; Falanga et al., 2018; Guarnieri et al., 2015; Galdiero et al., 2015). We also conjugated a targeting sequence falGea, which is able to recognize the overexpressed epidermal growth factor receptor and the epidermal growth factor receptor variant III (EGFR and EGFRvIII) (Gan et al., 2013; Mansour et al., 2022). Both gH625 and falGea peptides were conjugated to EPA-NPs through a linker made of Gly-Gly-Gly, while a C-terminal lysine residue was used for the attachment of EPA crucial for EPA-NPs formation. All peptides were conjugated to EPA, which is essential for the hydrophobic interactions that drive the formation of EPA-NPs.

The drug loading (DLC) can be directly read out from our calibration line, Supplementary material Fig. S10. In particular, we obtained a DLC of 13.42% for Doxorubicin and 8.83% for Pemetrexed.

To study cellular internalization through confocal microscopy studies, the N-terminus of gH625 was also labeled with Rhodamine B (Rho). We used two drugs Dox and PEM; Dox was used as a fluorescent model drug to facilitate nanoparticle uptake and intracellular distribution studies as well as preliminary cytotoxicity evaluation in HeLa cells, whereas PEM was selected for its clinical relevance in mesothelioma therapy. In particular, in both constructs (Dox-EPA and PEM-EPA), the drug is conjugated to EPA through a structured linker comprising a lysine residue followed by a tri-glycine spacer, as for the other constructs, and the MMP-9 cleavable peptide sequence “PLGSYL” (Fig. 1). This modular design enables selective drug release in the tumor microenvironment, where MMP-9 is overexpressed across multiple molecular cancer subtypes. The peptide linker is stable under acidic conditions; in fact, it was synthesized using solid-phase protocols and cleaved during resin treatment with 95% trifluoroacetic acid. The drug is then covalently attached in solution, and the resulting compound is purified by HPLC under acidic conditions. We also synthetized the peptide bearing Dox without the proteolytic cleavage sequence (Dox-EPA-NC), which we used as control in our experiments, as it did not release the drug within cells. The inclusion of the MMP-9 recognition sequence, combined with acid stability, ensures drug release primarily at the pathological site, enhancing therapeutic specificity and minimizing systemic side effects.

Regarding the synthesis, each peptide (Table 1) was synthesized on solid phase by employing the Fmoc/tBu strategy. We added the Fmoc-Lys(Mtt)-OH as the first amino acid, involved in the conjugation of the EPA on its side chain amino group after completing peptide assembly. At the end of the synthesis, each peptide was cleaved from the solid support, deprotected and purified by preparative HPLC and characterized by electrospray ionization (Supplementary material Fig. S2-S8). The Dox conjugation was performed in solution between the free thiol group on the cysteine side chain of the pure peptide and the maleimide moiety on Dox-EMCH. The reaction was confirmed by NMR spectroscopy (Supplementary material Fig. S9, panel A). One-dimensional NMR proton spectrum of Dox-EPA-NC showed resonances of both Dox and peptide moieties. The presence of the amides NHs of Gly and Lys was recognized by TOCSY spectra, where cross-peaks with their spin systems occurred. Chemical shift assignments were achieved by 2D spectra analysis. To estimate the degree of functionalization of the peptide with Dox, the integrals of unambiguous signals belonging to each moiety were compared. Signals at 4.08 ppm for Dox (4 OCH _3_, 13C 57.0 ppm) and at 2.01 ppm for peptide (COCH_3_, 13C 20.7 ppm) were chosen. Their comparison reveals an excess of Dox around 1.4. As regards Dox-EPA, NMR spectra showed clear signals of Dox and few weak signals of the peptide moiety, supporting the conjugation of the Dox to the peptide. The differences in the number of peaks revealed may be related to the fact that the peptide portion bound to the long unsaturated fatty acid does not enter as well into solution as Dox.

The PEM conjugation was performed on the solid phase. Firstly, the two carboxylic acid groups in PEM were protected as dimethyl esters (PEM(OMe)₂), and then it was added on the side chain of the glutamic acid using specific coupling reagents. After the precipitation and purification, the conjugation was confirmed by NMR spectroscopy. The 1D ^1^H NMR spectrum of PEM(OMe)₂-EPA shows resonances of both the peptide and PEM moieties, with the latter being more intense. The assignments were based on 2D TOCSY, NOESY, and HSQC spectra. The degree of peptide functionalization with PEM(OMe)₂-EPA was estimated by comparing the integrals of selected signals: at 7.25 ppm for PEM (two protons, integral 1.00) and at 0.84 ppm for leucine methyl groups in the peptide (six protons, integral 0.48). Moreover, after removal of dimethyl ester the PEM-EPA identity was confirmed by 1D ^1^H NMR analysis (Supplementary material Fig. S9 Panel B).

### 3.2 Mechanistic insights into the internalization of Dox-conjugated

#### 3.2.1 Construction and characterization of Dox-EPA-NPs

**Fig. 2:**
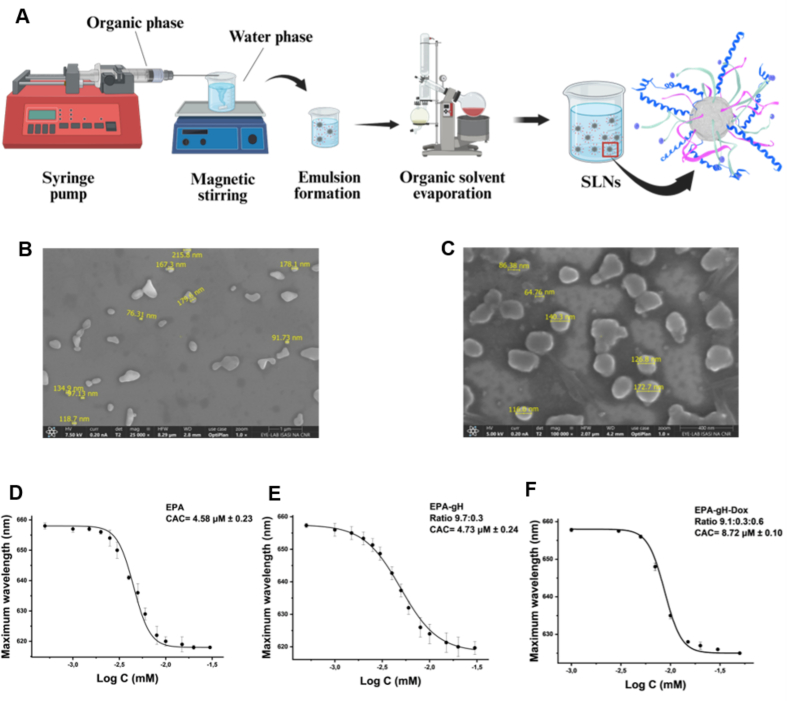
Description of the nanoemulsion solvent evaporation method (NSE) for the synthesis of EPA-NPs (A). Panel B—C SEM images of EPA-gH (B, scale bar = 1 μm) and EPA-gH-Dox (C, scale bar = 400 nm). Panels D-E–F report the CAC for EPA (D), EPA-gH (E), EPA-gH-Dox (F), wavelength corresponding to the maximum fluorescence emission of Nile red was plotted as a function of concentration and fitted using the sigmoidal Boltzmann equation (OriginPro Program for graphs). (For interpretation of the references to colour in this figure legend, the reader is referred to the web version of this article.)

We initially focused on identifying the optimal strategy to obtain nanoparticles composed of EPA with dimensions suitable for therapeutic applications. The size of EPA-NPs can change significantly because of EPA aggregation. Multiple approaches were evaluated, and in our early attempts, we produced EPA-NPs with excessively large dimensions (>400 nm, Supplementary Material, Table S1) using the standard self-assembly procedure, which involves film formation followed by hydration (Lombardi et al., 2019). Thus; we employed the nanoemulsion-solvent evaporation (NSE) method (Gomaa et al., 2022), a well-established technique for the preparation of drug delivery systems (Fig. 2, panel A). In this approach, an organic solvent containing the lipid phase is emulsified into an aqueous phase, resulting in the formation of nanoscale droplets. Then, the organic solvent is evaporated under reduced pressure at room temperature, yielding stable nanoparticles. This method ensures precise control over particle size and enhances solubility. Using this methodology, we were able to obtain EPA-NPs at concentrations up to 5000 μM for formulations composed solely of EPA (Supplementary material Table S1).

We employed dynamic light scattering (DLS) to measure the dimensions of EPA-NPs at concentrations ranging from 10 μM to 5000 μM, as reported in Table S2 (Supplementary material). Our results demonstrated that the EPA-NPs dimensions remained stable even at higher concentrations using the applied protocol. Furthermore, we analyzed the size of EPA-NPs at 5000 μM over different time points and observed a slight decrease in size and an increase in the Polydispersity Index (PdI) only after 30 days, indicating preserved structural integrity and consistent size distribution throughout the storage period. The presence of gH625 and Dox on the surface of the EPA-NPs can influence aggregation and lead to variations in nanoparticle properties. To explore this, we employed the NSE method to prepare EPA-NPs decorated with gH625 and Dox and characterized their dimensions. We evaluated different EPA:gH-EPA formulations containing up to 10% gH625 (EPA:gH-EPA = 9:1), monitoring changes in size and PdI at EPA-NPs concentrations ranging from 10 to 350 μM, the highest concentration used in the biological experiments for the preparation of stock solutions. The data presented in Table 2 clearly demonstrate that the presence of gH625 affects particle size. Specifically, EPA-NPs decorated with 35 μM (10%) of gH625 (the highest concentration tested) had a diameter size of 350.3 ± 16.8 nm, whereas reducing the gH625 concentration to 10.5 μM (3%) resulted in a smaller particle diameter of 193 ± 5 nm. For this reason, we selected the 3% gH625 for further functionalization with Dox.

**Table 2:** Characterization of different formulation by DLS analysis.

Formulation	Lipid and peptide ratio	Concentration (μM)	Size d. (nm)	PdI
EPA	–	350	390 ± 2	0.23 ± 0.04
EPA-gH	EPA: gH-EPA = 9:1	350	350 ± 1	0.23 ± 0.04
EPA-gH	EPA: gH-EPA =9.7:0.3	350	193 ± 5	0.26 ± 0.02
EPA-gH-Dox-NC	EPA: gH-EPA: Dox-EPA-NC = 9.1: 0.3: 0.6	350	140 ± 2	0.19 ± 0.01
EPA-gH-Dox	EPA: gH-EPA: Dox-EPA = 9.1: 0.3: 0.6	350	197 ± 3	0.12 ± 0.02

The next step involved the formulation of EPA-gH-Dox containing both gH625 and Dox. We selected a 6% ratio of Dox-EPA-NC or Dox-EPA for the formulation, as this composition proved to be the most suitable to prevent excessive aggregation of the system. Higher peptide concentrations led to increased particle clustering, whereas the 6% formulation maintained better colloidal stability. This choice was supported by preliminary confocal microscopy observations, which showed an excessive aggregation at the highest concentration (Supplementary Materials Fig. S11). Specifically, the nanoparticle formulation EPA-gH-Dox included EPA, gH-EPA, and Dox-EPA in a 9.1:0.3:0.6 M ratio, respectively. To assess the effectiveness of the on-demand drug release strategy, we also prepared EPA-gH-Dox-NC, as a control for cellular experiments, using the same ratio.

The particle size remained unchanged upon the addition of Dox to the formulation (EPA-gH-Dox). Specifically, the particle diameter for EPA-gH-Dox was 197 nm (Table 2), while the size of EPA-gH-Dox-NC, was 140 nm with a PdI of 0.19 ± 0.01.

The morphology and size of both EPA-gH and EPA-gH-Dox, formulated at molar ratios of 9.7:0.3 and 9.1:0.3:0.6, respectively, were evaluated using Scanning Electron Microscopy (SEM). The average diameter of both EPA-gH and EPA-gH-Dox nanoparticles ranged from 120 to 180 nm (Fig. 2, panels B—C, respectively), in line with DLS measurements, which provided a diameter of approximately 200 nm for both formulations, as also reported in Table 2. The particle size of EPA-gH-Dox-NC is approximately 140 nm, likely due to improved formulation compaction compared to EPA-gH-Dox, where the MMP-9 sequence is presumably exposed to the aqueous environment.

To identify the concentration at which the system aggregates, the critical aggregation concentration (CAC) was determined using Nile Red (NR) as a fluorescent probe across a concentration range from 0.5 μM to 30 μM. Nanoparticle formulations were added to NR solution and recorded a blue shift in NR emission fluorescence, which indicates the dye transfer into a more hydrophobic environment, such as the aggregates. The measurements were performed in triplicate, and CAC values were obtained by fitting the data to the Boltzmann equation (Fig. 2, panels D-*E*—F). The CAC value for EPA was 4.58 ± 0.23 μM, reflecting strong hydrophobic interactions (Fig. 2, panel D). The presence of gH625 in EPA-gH did not significantly affect the CAC, which was 4.73 ± 0.24 μM (Fig. 2, panel E). In contrast, the incorporation of both gH625 and Dox altered the nanoparticle surface properties, resulting in an increase in the CAC to 8.72 ± 0.10 μM (Fig. 2, panel F). Additionally, zeta potential analysis indicated that the surfaces of both EPA-gH and EPA-gH-Dox, were slightly positively charged. For EPA-gH-Dox, the measured zeta potential was approximately +1.3 mV (Table 3). This, together with the size data, demonstrates that the EPA-NPs are well-defined in both dimensions and surface charge, making them suitable for therapeutic applications. The slightly positive zeta potential value is important for preventing excessive aggregation while minimizing potential toxicity associated with highly positively charged particles.

**Table 3:** Critical aggregation concentration (CAC) values for each formulation.

Formulation	Lipid and peptide ratio	CAC (μM)	Zeta Potential (mV)
EPA	–	4.58 ± 0.23	+ 0.6 ± 0.1
EPA-gH	9.7:0.3	4.73 ± 0.24	+ 4.2 ± 0.5
EPA-gH-Dox	9.1: 0.3: 0.6	8.72 ± 0.10	+ 1.3 ± 0.6

The presence of gH625 on the EPA-NPs surface was confirmed by Circular Dichroism (CD) spectroscopy (Supplementary material Fig. S12). We previously demonstrated that gH625 transitions from a random coil to a helical conformation when interacting with membrane bilayers (Ferraro et al., 2021). We analyzed its secondary structure bound to EPA-NPs in phosphate buffer and 20% of 2,2,2-trifluoroethanol (TFE) at 8 μM, where TFE reduces solvent polarity and facilitates the stabilization of secondary structures. In water, gH625 bound to EPA-NPs was aggregated, but in 20% TFE, it adopted an α-helical conformation, crucial for membrane interaction and internalization in delivery processes.

#### 3.2.2 Analysis of the stability of EPA-NPs

The stability of fully formulated EPA-gH-Dox was assessed at 350 μM, the concentration of stock solutions used for biological assays. The formulation was stored at +4 °C for up to 30 days. EPA-gH-Dox demonstrated good stability, with the particle diameter remaining within the 150–200 nm range and showing no significant changes over the storage period (Table 4).

**Table 4:** Time stability of EPA-gH-Dox.

Time (days)	Size (d. nm)	PdI
0	163 ± 9	0.19 ± 0.04
7	170 ± 2	0.02 ± 0.01
14	156 ± 1	0.10 ± 0.02
21	158 ± 5	0.21 ± 0.01
28	143 ± 3	0.19 ± 0.02

The stability of EPA-NPs under various environmental conditions was evaluated, including dilution, ionic strength, and pH (Supplementary material Fig. S13). We monitored the effects of these parameters on EPA-NP size using dynamic light scattering (DLS). To assess dilution effects, EPA-NPs were initially prepared at 350 μM and then gradually diluted. Ionic strength stability was tested by adding NaCl in concentrations ranging from 1 mM to 5 mM. For pH stability, EPA-NPs were tested at pH values of 3, 7, and 10 (Supplementary material Fig. S132). No significant changes in size were observed under any of these conditions, supporting our hypothesis that the EPA-NPs remain stable and do not disaggregate under the external changes tested.

EPA-gH-Dox stability in fetal bovine serum was also evaluated. DLS measurements did not show significant changes in peak intensity, showing only a slight increase in size after 48 h. However, at 72 h, the system was unstable, with the appearance of a peak size of approximately 39 ± 2 nm and a PdI of 1.00, indicative of a high polydispersity index (Table 5).

**Table 5:** Serum stability of EPA-gH-Dox.

Time (hours)	Size (d. nm)	PdI
0	154 ± 1	0.10 ± 0.06
3	162 ± 6	0.13 ± 0.02
24	129 ± 2	0.31 ± 0.01
48	167 ± 7	0.41 ± 0.05

#### 3.2.3 Uptake of Rho-gH-EPA and EPA-gH-Dox

**Fig. 3:**
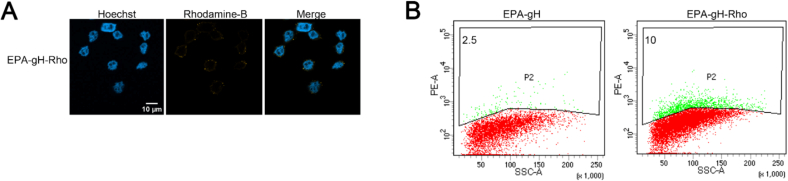
**A)** 2D visualization of EPA-NPs decorated with Rho-gH-EPA; Rhodamine-B is yellow while nucleus is colored in blue with Hoechst. Here, we show a representative image from three independent experiments. **B)** Cytofluorimetric analysis of Rhodamine B signals. One of two independent experiments is shown. (For interpretation of the references to colour in this figure legend, the reader is referred to the web version of this article.)

The cellular uptake of the formulation EPA-gH by HeLa cells *in vitro* was assessed *via* confocal fluorescence microscopy using the Rhodamine B-labeled gH-EPA peptide in the formulation. EPA-NPs were prepared at 350 μM, with 20 μM of Rho-gH-EPA, and diluted to 175 μM with a final concentration of 10 μM of Rho-gH-EPA for the experiments. After 1 h of incubation, cells were washed, fixed and imaged. 2D confocal images (Fig. 3) revealed EPA-NPs localized in the cytoplasmic, perinuclear, and nuclear regions.

**Fig. 4:**
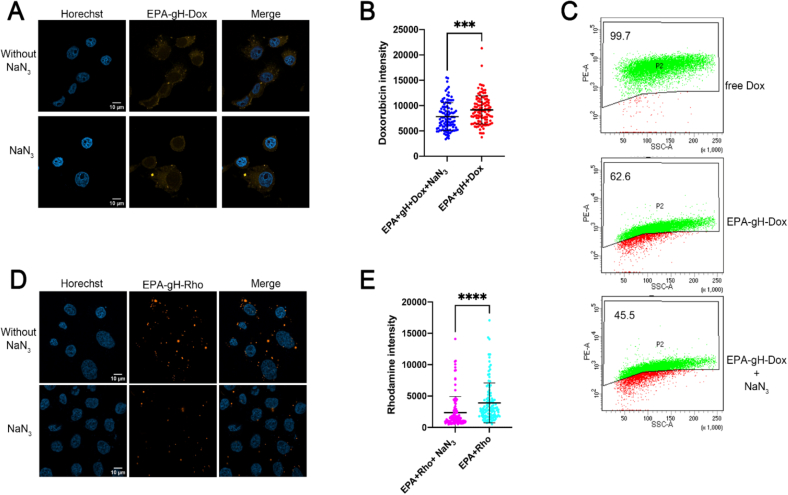
The cellular uptake of EPA-gH-Dox and Rho-gH-EPA evaluated on HeLa cells **A)** Representative images of HeLa cells pretreated with NaN_3_ at 40 μM for thirty minutes at 37 °C, followed by incubation with EPA-gH-Dox for additional three hours. At the end of incubation time, HeLa cells were fixed and analyzed by confocal microscopy. **B)** Dox quantification analysis was carried out with Fiji software. Results are presented as the mean ± standard deviation (SD) from three independent experiments. Statistically significant differences are indicated as **** *p* < 0.0001. We used Welch's-corrected Student *T*-Test for the statistical analysis to avoid distortion due to the heteroscedasticity of the examined sample. Around thirty cells were analyzed for each condition. **C)** Cytofluorimetric analysis of doxorubicin levels following treatment with … for two hours. At the end of the incubation period, cells were fixed with 4% paraformaldehyde and analyzed**. D)** HeLa cells were treated as in A followed by analysis of Epa-gH-Rhodamine signal. **E)** Statistical analysis was performed as in B.

Additionally, the cellular internalization mechanism of Rho-gH-EPA and EPA-gH-Dox was evaluated in HeLa cells by performing the inhibition experiment. We previously demonstrated that the peptide gH625 favors the nanosystem internalization across membranes mainly through a translocation mechanism and involving only partially the endocytosis pathway (Bellavita et al., 2024). HeLa cells were pretreated with NaN_3_ at 40 μM for thirty minutes at 37 °C, followed by incubation with EPA-gH-Dox for additional three hours. At the end of incubation time, HeLa cells were fixed and analyzed by confocal microscopy. Subsequently, Dox quantification analysis was carried out with Fiji software. As observed in Fig. 4, the treatment with sodium azide (NaN_3_) to inhibit the active pathway had little effect on Rho-gH-EPA and EPA-gH-Dox, indicating that active pathway partially contributes to their cellular internalization. Overall, these findings suggest that gH625 predominantly promotes membrane crossing through a translocation mechanism.

#### 3.2.4 Dox release and biocompatibility of EPA-gH-Dox NPs

Dox release from EPA-gH-Dox and EPA-gH-Dox-NC EPA-NPs were evaluated by UV–Vis spectroscopy following incubation with MMP-9 for 30, 60, and 120 min. The enzyme recognizes the sequence PLGSYL and cuts the bond between Gly and Ser aminoacids (Guarnieri et al., 2015). In particular, the enzyme was pre-activated with 4-aminophenylmercuric acetate (APMA) for 3 h at 37 °C before being added to the nanoparticles After the incubation, the samples were centrifuged to remove the nanoparticles, and Dox absorbance was measured at 480 nm. As shown in Table 6, approximately 50 ± 2% of Dox was released by EPA-gH-Dox after 60 min, reaching 80 ± 2% within 120 min; while for EPA-gH-Dox-NC no significant release was observed. Moreover, as control, we also performed the same experiments without the enzyme and did not observe any Dox release (data not shown).

**Table 6:** The percentage of Dox released at different time points.

Time (min)	Dox released EPA-gH-Dox (%)	Dox released EPA-gH-Dox-NC (%)
30	10 ± 1	1 ± 1
60	50 ± 2	1 ± 1
120	80 ± 1	2 ± 1

We also assessed Dox release in HeLa cells following uptake of EPA-gH-Dox and its control counterpart, EPA-gH-Dox-NC, which lacks the proteolytic sequence.

**Fig. 5:**
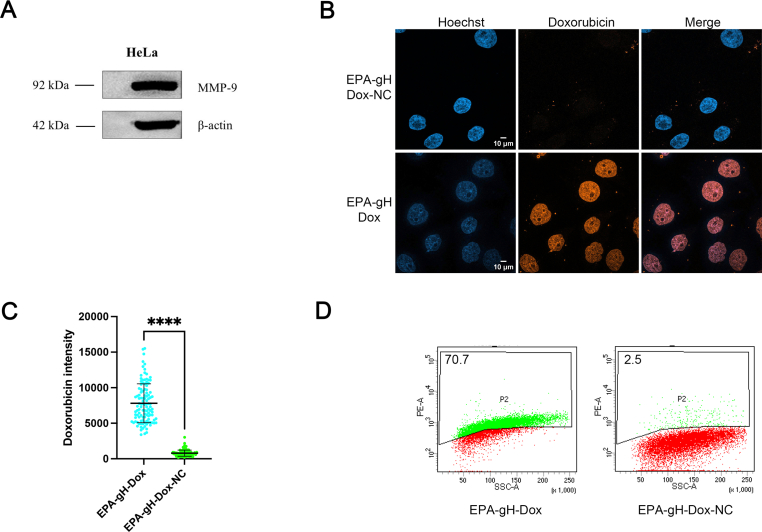
**A)** Western blotting analysis of MMP-9 expression in HeLa cells. **B)** Confocal microscopy images (panel B) show the absence of Dox release in the non-cleavable system (EPA-gH-Dox-NC), while drug release was detected in the presence of the cleavable sequence (EPA-gH-Dox). **C)** Quantification analysis, of Doxo signal by confocal microscopy and Fiji software. We monitored thirty cells for each condition, from three independent experiments reporting the SD. **** *p* < 0.001. The statistical analysis was carried out with Welch's-corrected Student *t*-test, to reduce the effect of heteroscedasticity-related distortions in the analyzed sample. **D)** Cytofluorimetric analysis of Doxorubicin signal in HeLa cells in the presence or absence (NC) of MMP9 cleavable sequence. Here we reported one of the three independent experiments.

The Western blot analysis was also performed to confirm MMP-9 expression in HeLa cells. As expected, MMP-9 was detected at 92 kDa, while β-actin, used as the loading control, appeared at 42 kDa (Fig. 5, panel A). Following confirmation of MMP-9 presence, we evaluated *in vitro* Dox release from EPA-gH-Dox-NC and EPA-gH-Dox.

In the absence of the MMP-9 cleavable linker (EPA-gH-Dox-NC), Dox intensity signal was reduced in comparison to the EPA-gH-Dox, as observed also in quantification analysis of Dox signal by confocal microscopy and flow cytometry analysis (Fig. 5, panels B—D). In contrast, the presence of the cleavage sequence allowed the release of Dox, which was free to reach its characteristic nuclear localization (Fig. 5, panel B). This indicates efficient intracellular drug release mediated by the MMP-9 cleavable sequence, whereas the non-cleavable system retains the drug associated with NP, resulting in low signal. Additionally, as reported in Fig. 5D, flow cytometry analysis confirms these findings, showing a markedly higher percentage of Dox-positive cells for EPA-gH-Dox (∼71%) compared to EPA-gH-Dox-NC (∼2.5%).

**Fig. 6:**
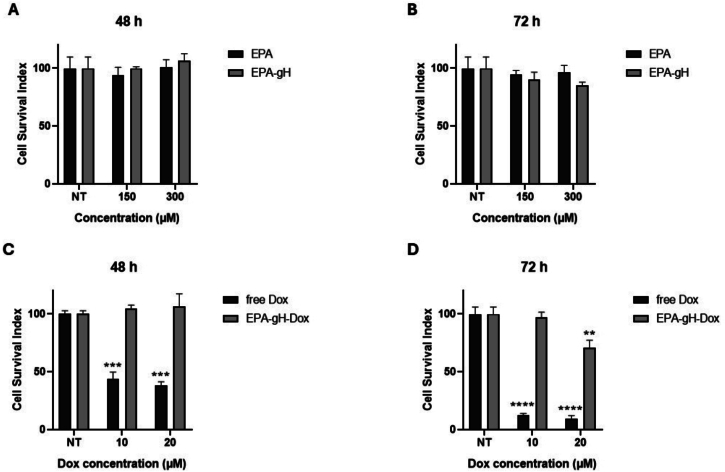
**A, B)** Cell viability of HaCaT cells at 48 (A) and 72 h (B) for EPA alone and the formulation EPA-gH. **C,D)** Cell viability of HaCaT cells at 48 (C) and 72 h (D) for the formulation EPA-gH-Dox and free Dox.

To assess the impact of the carrier, EPA and EPA-gH, on healthy cells that constitutively express MMP-9, we measured the cell survival index in HaCaT cells at 48 and 72 h. As shown in Fig. 6 (panels A-B), the carriers do not reduce viability up to the highest concentration tested (300 mM).Then we compared the activity of EPA-gH-Dox with that of free Dox in HaCaT cells at 48 and 72 h to confirm that Dox is not significantly released in cells where the MMP-9 is constitutively expressed. As shown in Fig. 6, panels C—D, free Dox exhibited marked toxicity as early as 48 h, while EPA-gH-Dox did not affect cell viability. At 72 h, the toxicity of free Dox increased, whereas EPA-gH-Dox remained non-toxic up to 150 μM; while minimal toxicity was only observed at 300 μM of EPA-gH-Dox. These findings support that EPA-NPs are well tolerated by healthy cells, likely due to the low MMP-9 levels, limiting Dox release.

**Fig. 7:**
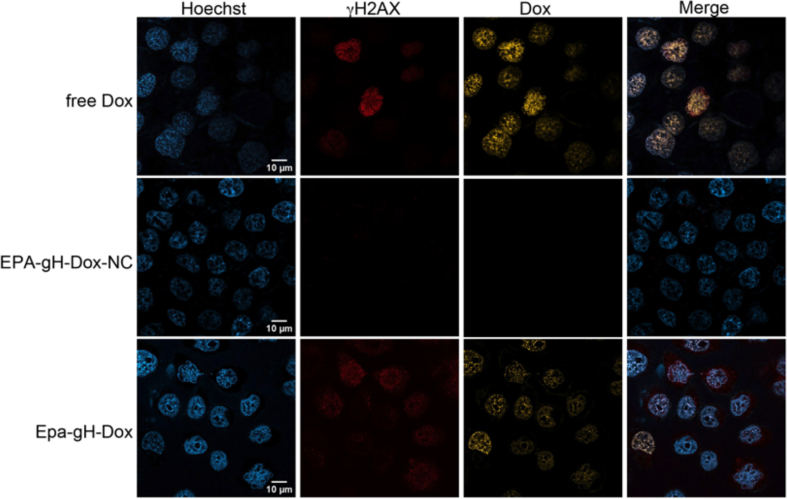
Confocal microscopy images of HeLa cells treated with free-Dox, EPA-gH-Dox-NC and EPA-gH-Dox. The nucleus is colored blue with Hoechst, histone phosphorylation in red, Dox fluorescence in yellow. The rightmost column shows merged images of the three channels. (For interpretation of the references to colour in this figure legend, the reader is referred to the web version of this article.)

To further confirm both internalization and intracellular release of Dox in HeLa cells, we assessed DNA damage using an immunofluorescence assay targeting γ-H2AX, a marker for genotoxic stress induced by ATM-mediated phosphorylation of histone H2AX at serine 139. Cells were treated with EPA-gH-Dox and its control EPA-gH-Dox-NC (lacking the MMP-9-cleavable sequence), both containing 3% gH625 (5.25 μM) and 6% Dox (10.5 μM), alongside free Dox as a control. After a 3 h incubation, sufficient for MMP-9 mediated cleavage and drug release, γ-H2AX foci were detected using a specific antibody, serving as an indicator of DNA damage caused by Dox activity (Fig. 7).

In cells treated with EPA-gH-Dox-NC, Dox localization in the nucleus was not clearly detectable. Nonetheless, some red γ-H2AX foci, indicative of DNA damage through histone phosphorylation, were present likely due to limited Dox release following partial disassembly of the EPA-NPs. In contrast, EPA-gH-Dox showed clear nuclear localization of Dox and more extensive DNA damage. This is attributed to the overexpression of MMP-9 and the consequent release of Dox. Compared to free Dox, the observed DNA damage was less intense, reflecting a controlled, gradual release. This process relies on the initial EPA-NP internalization *via* gH625, followed by enzyme-triggered Dox release.

Dox release led to histone damage, seen as red γ-H2AX foci (Fig. 7). However, the level of histone phosphorylation was lower than with free Dox, reflecting the controlled, gradual release from EPA-NPs over time. In this system, the Dox showed predominantly nuclear localization, but some cytosolic presence was also noted, particularly near the membrane, suggesting that the process of drug release was still going on. In addition, also the Uv/Vis studies have shown that system incubation with pre-activated MMP-9 results in a release of about 80% of the drug after 2 h, indicating that the Dox has not yet been fully released and is gradually released (as reported above in Table 5).

#### 3.2.5 Cell proliferation assays

**Fig. 8:**
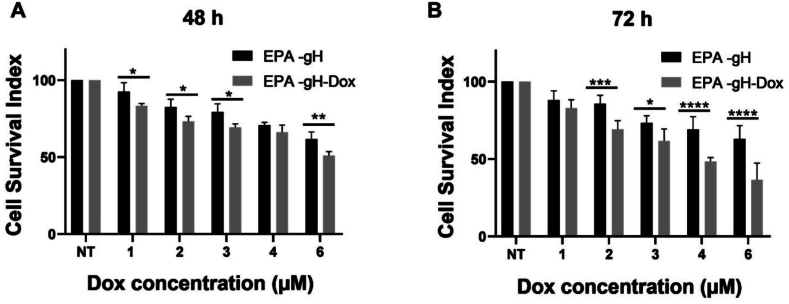
**A,B)** Cytotoxicity analysis of HeLa cells treated with EPA-gH, and EPA-gH-Dox at 48 h (A) and 72 h (B). The graphs represent means ± standard deviation of 3 independent experiments. Statistically analysis was performed by using one-way repeated measures ANOVA to compare all groups *versus* control and significant differences are indicated with: *P-value <0.05, ***P-value <0.01, **P-value<0.01.

Moreover, the Dox release and its cytotoxic effect were further investigated on HeLa cells treated with EPA-gH and EPA-gH-Dox for 48 h and 72 h. All treatments give statistically significant results showing a reduction in cell proliferation compared to the control already at 48 h. Also EPA-gH exhibited some toxicity at 48 and 72 h, suggesting that the delivery platform developed holds promise for cancer applications (Tsugane, 2021). The EPA-gH-Dox system exhibited a significantly greater reduction in cell viability at Dox concentrations of 1, 2, 3, 4, and 6 μM compared to EPA-gH alone at same Dox concentration, confirming the cytotoxic effect of the released drug. At 6 μM and 72 h, treatment with EPA-gH-Dox resulted in ∼40% cell viability, whereas free Dox reduced viability to ∼10%. (Fig. 8).

### 3.3 EPA-NPs decorated with PEM tailored for mesothelioma

#### 3.3.1 Biophysical characterization of EPA-NPs decorated with PEM (EPA-gH-TP-PEM)

Following successful validation in a model cell system (HeLa cells), the nanosystem was subsequently evaluated in mesothelioma as a proof of concept, a highly aggressive cancer with limited treatment options. To address this, we formulated and characterized the formulation EPA-gH-TP-PEM specifically designed for mesothelioma treatment, incorporating the targeting peptide falGea (TP). The biophysical characterization, including the optimization of the nanoparticle composition and evaluation of key physicochemical properties such as size, PdI and surface charge, were performed to ensure their stability and suitability for biological testing in mesothelioma models.

**Fig. 9:**
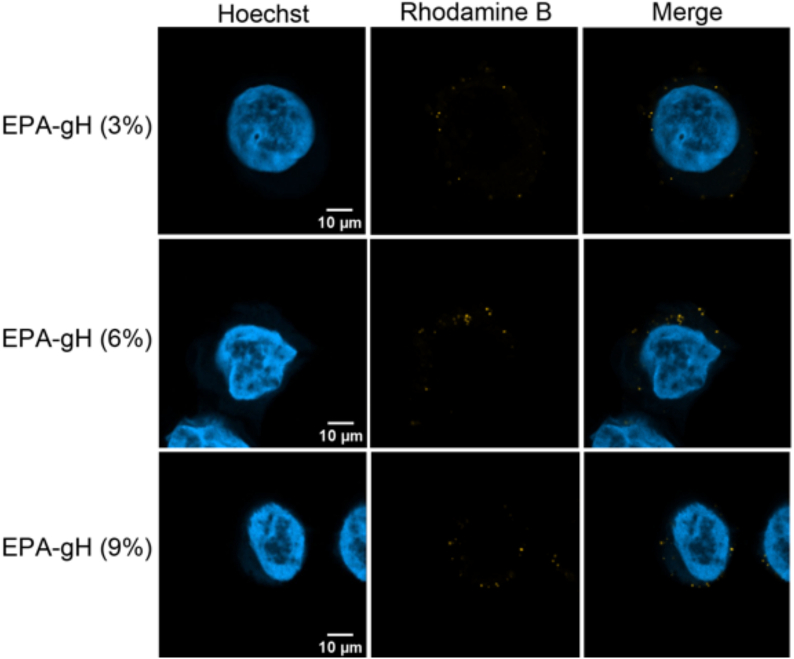
Confocal microscopy images of cells treated with nanoparticles containing 3%, 6%, and 9% of EPA-gH.

To further advance our previous formulation, we engineered EPA-gH-TP-PEM by integrating the membrane-active peptide gH625, the targeting peptide falGea, and the chemotherapeutic agent PEM at a molar ratio of 9.09:0.3:0.6:0.01. The optimal amount of gH625 was evaluated using confocal microscopy by testing formulations containing 3%, 6%, and 9% of the peptide (Fig. 9). Image analysis revealed that 6% gH625 provided the best balance between cellular visualization and prevention of nanoparticle aggregation.

Based on previous findings, 6% was also identified as the optimal concentration for the targeting peptide falGea to enhance cellular internalization (Bellavita et al., 2025). Lastly, the effective concentration of PEM was determined by assessing its half-maximal inhibitory concentration (IC50), which was found to be 50 nM.

**Fig. 10:**
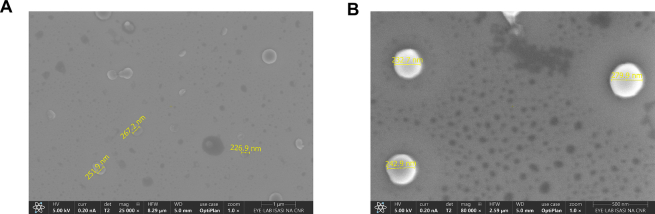
**A,B)** SEM images of EPA-gH-TP-PEM at 25000× magnification (A, scale bar = 1 μm) and 80,000× magnification (B, scale bar = 500 nm).

The particle size of the formulation EPA-gH-TP-PEM was 228 ± 7 nm, with a PdI of 0.16 ± 0.03 (Table 7). Morphology and size were further assessed using SEM, revealing an average diameter ranging from 220 to 270 nm (Fig. 10), consistent with the DLS data.

**Table 7:** Characterization of different formulation by DLS analysis.

Formulation	Lipid and peptide ratio	Concentration (mM)	Diameter (nm)	PdI
EPA-gH	EPA: gH-EPA = 9.4:0.6	250	234 ± 3	0.37 ± 0.10
EPA-gH-TP	EPA: gH-EPA: falGea-EPA = 9.1:0.6:0.3	250	124 ± 19	0.18 ± 0.01
EPA-gH-TP-PEM	EPA: gH-EPA: falGea-EPA: PEM-EPA = 9.09: 0.3: 0.6:0.01	250	228 ± 7	0.16 ± 0.03

**Fig. 11:**
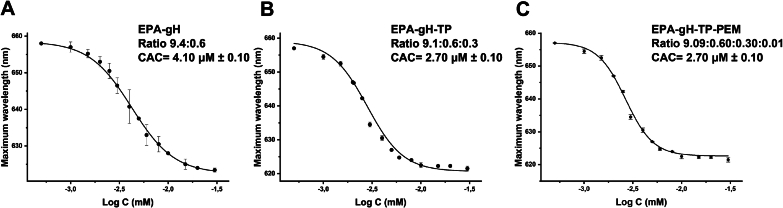
**A,B,C)** CAC for EPA-gH (A), EPA-gH-TP (B), EPA-gH-TP-PEM (C), wavelength corresponding to the maximum fluorescence emission of Nile red was plotted as a function of concentration and fitting using the sigmoidal Boltzmann equation (OriginPro Program for graphs). (For interpretation of the references to colour in this figure legend, the reader is referred to the web version of this article.)

The CAC measurements were performed in triplicate, for all the formulations. The CAC for EPA-gH was found to be 4.10 ± 0.10 μM (Fig. 11, panel A), while the addition of the targeting peptide (TP) resulted in smaller nanoparticles and a lower CAC which was found to be 2.70 ± 0.10 μM (Fig. 11, panel B). Furthermore, the subsequent addition of the drug did not modify the CAC. Notably, these results highlight the effect of surface complexity on aggregation behavior.

We also determined the zeta potential of the obtained EPA-NPs, which was found to be slightly positive for all formulations (Table 8). This feature helps limit excessive aggregation while reducing the risk of toxicity typically associated with highly positive surface charges. Indeed, the obtained EPA-NPs are well-defined in both dimensions and surface charge, making them suitable for therapeutic applications.

**Table 8:** Critical aggregation concentration (CAC) values for each formulation.

Formulation	Lipid and peptide ratio	CAC (μM)	Zeta Potential (mV)
EPA-gH	9.4:0.6	4.10 ± 0.11	+ 5.9 ± 0.6
EPA-gH-TP	9.1:0.6:0.3	2.70 ± 0.12	+ 6.9 ± 0.2
EPA-gH-TP-PEM	9.09: 0.30: 0.60:0.01	2.70 ± 0.13	+ 3.6 ± 0.1

The stability was assessed at 250 μM, the concentration of the stock solutions used for biological assays in the mesothelioma model system. EPA-gH-TP-PEM was shown to be stable, although a decrease in stability was observed at 72 h, as indicated by changes in particle size (Supplementary material Fig. S14). Importantly, these findings indicate that the current formulation is suitable for short-term biological studies and will need to be optimized to meet the stability requirements for prolonged systemic circulation *in vivo*. Furthermore, the effects of dilution, ionic strength, and pH on EPA-NPs size were evaluated using DLS. To assess dilution effects, EPA-NPs were initially prepared at a concentration of 250 μM and subsequently diluted. Ionic strength stability was tested by adding NaCl at concentrations ranging from 1 mM to 5 mM and pH stability was evaluated at pH values of 3, 7, and 10. Across all tested conditions, no significant changes in EPA-NPs size were observed, indicating that the nanoparticles remained stable and did not undergo disaggregation in response to these external stimuli (Supplementary material Fig. S15).

#### 3.3.2 Uptake and cell proliferation assay for EPA-PEM

**Fig. 12:**
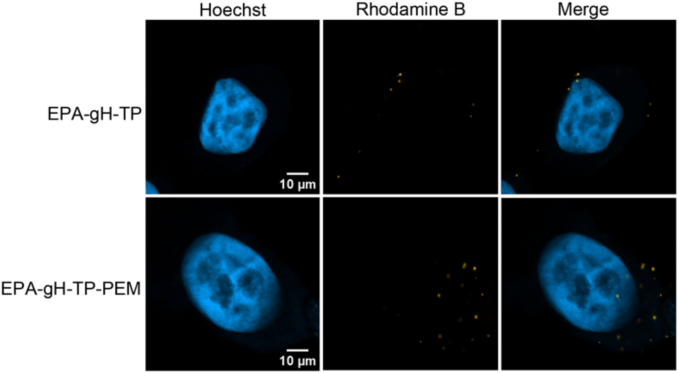
Confocal fluorescence microscopy images of MSTO-211H cells after 1 h incubation with the formulations EPA-gH-TP and EPA-gH-TP-PEM (100 μM, containing 6% Rho-gH-EPA). Fluorescence signal (yellow) indicates cellular uptake and localization of the EPA-NPs in the cytoplasmic, perinuclear, and nuclear regions. (For interpretation of the references to colour in this figure legend, the reader is referred to the web version of this article.)

The cellular uptake of EPA-gH-TP and the complete EPA-gH-TP-PEM system was evaluated *in vitro* by confocal fluorescence microscopy, using Rho-gH-EPA included in the formulation. EPA-NPs were prepared at a concentration of 250 μM, containing 6% Rho-gH-EPA, and diluted to 100 μM for the experiments. After 1 h of incubation with MSTO-211H cells, samples were washed, fixed, and imaged by confocal microscopy. 2D confocal images (Fig. 12) showed the internalization of both systems, with fluorescence localized in the cytoplasmic, perinuclear, and nuclear regions.

**Fig. 13:**
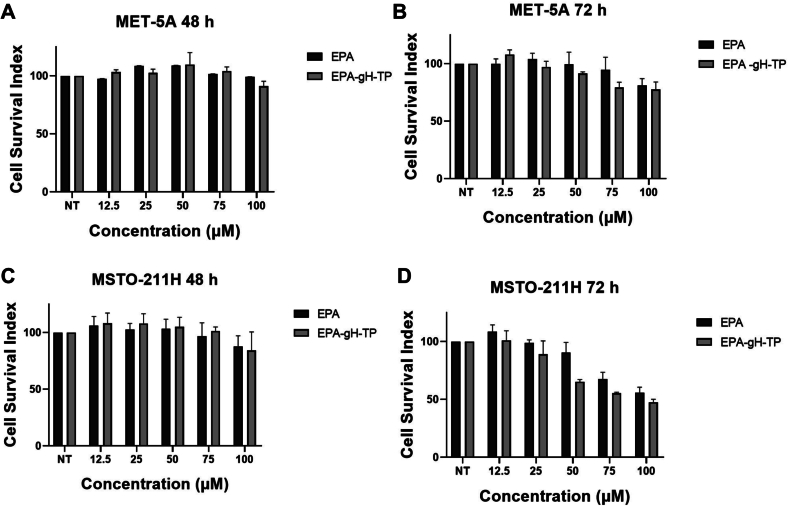
Cell viability of MET-5 A**(A,B)** and MSTO-211H **(C,D)** cells at 48 and 72 h for EPA and EPA-gH-TP.

The biocompatibility and the ability to inhibit proliferation were assessed using MET-5 A (non-tumoral mesothelial) and MSTO-211H (biphasic mesothelioma) cell lines. In MET-5 A cells, all formulations (EPA and EPA-gH-TP) exhibited no detectable cytotoxicity up to 100 μM at both 48 and 72 h, indicating excellent compatibility with healthy cells (Fig. 13, panels A–B).

In contrast, a distinct response was observed in MSTO-211H mesothelioma cells, where EPA-NPs led to a reduction in cell viability. Notably, EPA-gH-TP induced a 40% decrease in viability at 72 h (Fig. 13, panels C—D). These findings highlight the selective cytotoxicity of the nanosystem, demonstrating its safety in healthy cells while revealing that the carrier itself contributes to anti-cancer effects in malignant cells. This was a particularly important result as the EPA-NPs were designed to be selective for cancer cells and active also when the drug is not released on cancer cells.

**Fig. 14:**
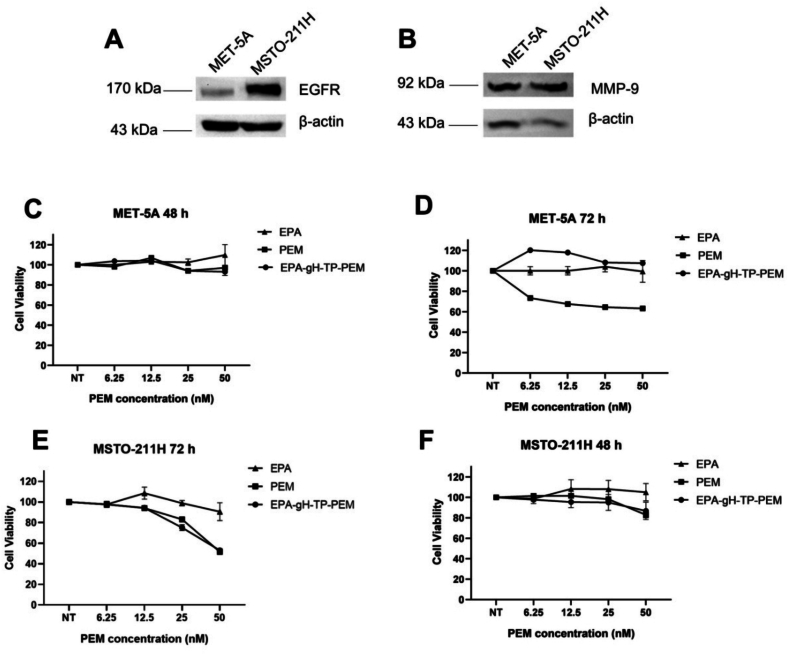
Western blot analysis of EGFR and MMP-9 expression in MET-5 A (non-tumoral mesothelial cells) and MSTO-211H (biphasic mesothelioma) cell lines **(A, B)** and cell viability of EPA-gH-TP-PEM and Pemetrexed on MET-5 A after 48 **(C, D)** and MSTO-211H after 48 h and 72 h **(E, F)**.

Then, we performed a Western blot analysis to confirm MMP-9 and EGFR expression in MET-5 A and MSTO-211H cell lines. As expected, EGFR and MMP-9 are overexpressed in the MSTO-211H tumor cell line compared to MET-5 A (Fig. 14, panels A-B). To establish the cytotoxicity profile, MET-5 A and MSTO-211H cells were treated with EPA-gH-TP-PEM and free PEM. All treatments give statistically significant results showing a reduction in cell proliferation compared to the control already at 48 h. In cell lines lacking EGFR and MMP-9 overexpression, EPA-gH-TP-PEM show no significant reduction in cell viability, suggesting limited drug release in the absence of MMP-9-mediated cleavage, while PEM treatment reduced cell viability to approximately 60–70% at the highest concentration (50 nM) after 72 h. Conversely, in the MSTO-211H cell line, after 72 h, EPA-gH-TP-PEM displays cytotoxicity comparable to the free drug, with a reduction of cell viability to ∼50–60% at 50 nM.

## 4 Conclusion

The development of biocompatible carrier materials is crucial for enhancing drug delivery. In oncology, the development of nanoparticles with pre-determined properties represents a promising new strategy, enabling controlled and targeted delivery of anticancer drugs with increased efficacy and reduced side effects. This study aimed to develop biocompatible EPA-NPs using EPA as a structural component. While previous research has demonstrated the synergistic activity of omega-3 fatty acids with drugs like Dox, this study is the first to report the development of a nanocarrier entirely composed of EPA, thereby minimizing any potential systemic toxicity associated with the carrier. EPA is primarily found in fatty fish and plays a crucial role in reducing inflammation, supporting cardiovascular and mental health. Moreover, toxicity issues commonly associated with nanocarriers are often linked to synthetic or non-biodegradable materials, accumulation of excipients, or chemically modified lipids. In contrast, EPA is a naturally occurring omega-3 fatty acid with a well-established safety profile and is readily metabolized through endogenous lipid pathways. The use of unconjugated EPA as the sole structural component is therefore expected to minimize long-term accumulation and reduce the risk of carrier-related toxicity. Future *in vivo* studies will be aimed at systematically evaluating biodistribution, clearance, and safety to further substantiate these assumptions.

Our results indicate that the developed EPA-NPs retain excellent biocompatibility as shown by the cytotoxicity studies on HaCaT cells, which clearly indicate that Dox is toxic for healthy cells while our developed EPA-NPs conjugated to Dox (EPA-gH-Dox) present no toxicity up to 72 h at the concentration useful for anticancer activity. Furthermore, the prepared EPA-NPs demonstrate similar efficacy compared to free Dox in inhibiting the viability of HeLa cells *in vitro*; indeed, the activity of our EPA-NPs is obtained because of the on-demand release of the drug, thus is not inducing any systemic toxicity. As a matter of fact, the control compound in which the Dox is covalently linked to the surface of the EPA-NPs without the proteolytic sequence does not show any toxicity both in HaCaT and HeLa cells, highlighting that the EPA-gH-Dox is active in cancer cells overexpressing the MMP-9 but not in healthy cells which only constitutively express the MMP-9. Furthermore, Dox release led to histone damage, which resulted lower than with free Dox, reflecting the controlled, gradual release from the EPA-NPs over time.

**Fig. 15:**
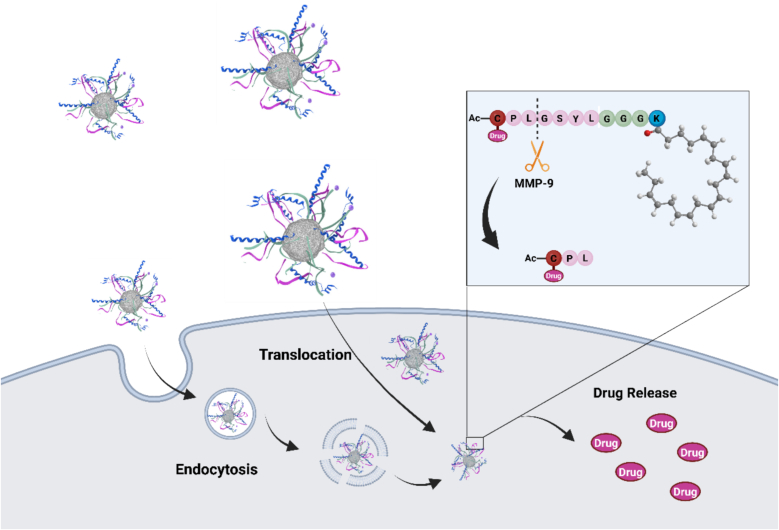
Schematic representation of omega-3-based EPA-NPs mechanism of action. The figure was created with BioRender.

Expanding these initial results, we subsequently employed mesothelioma cells to evaluate our EPA-NPs in a more clinically relevant and disease-specific setting. Specifically, we employed both a non-tumoral mesothelial cell line (MET-5 A) and a biphasic mesothelioma cell line (MSTO-211H) to assess the differential response. Our results confirmed that EPA-gH-TP, is non-toxic to healthy mesothelial cells while exhibiting cytotoxic activity against cancer cells. Importantly, when loaded with the chemotherapeutic agent (PEM), the EPA-NPs demonstrated an effect comparable to that of the free drug. Additionally, the formulation containing the gH625 peptide facilitating the translocation of EPA-NPs and their release from endocytic vesicles (Fig. 15), enabled an on-demand release of PEM, which is attributed to the enzymatic activity of MMP-9, overexpressed in the tumor microenvironment. This enzyme-responsive behavior supports the targeted and controlled drug release capabilities of our nanosystem. Given mesothelioma's aggressive progression and the scarcity of effective treatments, this approach provided an opportunity to showcase the versatility and translational potential of our nanosystem.

Additionally, EPA possesses itself anticancer activity while simultaneously reducing toxicity to healthy cells, resulting in a more effective and safer treatment approach.

In conclusions, the results demonstrate promising *in vitro* cytocompatibility, enzyme-responsive drug release, and anticancer activity. However, it should be noted that the studies conducted in 2D models are preliminary and inherently limited, as they do not fully recapitulate the complexity of the tumor microenvironment. The next steps will involve validation in more complex 3D systems and subsequent *in vivo* investigations to evaluate pharmacokinetics, biodistribution, tumor targeting, and overall therapeutic efficacy, as well as to address current limitations related to serum stability.

## CRediT authorship contribution statement

**Simone Braccia:** Formal analysis, Data curation, Conceptualization. **Luigi Alfano:** Formal analysis, Data curation, Conceptualization. **Maria Carmen Ragosta:** Formal analysis, Data curation. **Rosa Bellavita:** Formal analysis, Conceptualization. **Gabriella D'Auria:** Formal analysis, Data curation. **Emanuela Esposito:** Data curation. **Federica Donadio:** Data curation. **Sara Palladino:** Formal analysis, Data curation. **Alessandro di Vaio:** Data curation. **Rosa Camerlingo:** Methodology, Formal analysis. **Lucia Falcigno:** Formal analysis, Data curation. **Annarita Falanga:** Formal analysis, Conceptualization. **Michelino de Laurentiis:** Resources, Investigation, Conceptualization. **Antonio Giordano:** Resources, Investigation, Conceptualization. **Stefania Galdiero:** Investigation, Conceptualization, Writing – review & editing, Writing – original draft.

## Declaration of competing interest

The authors declare no competing interest. The authors Braccia S, Bellavita R, Falanga A, Giordano A, Galdiero S, hold a patent for the design and development of EPA- based nanoparticles (IT patent no.: 102025000026059).

## Data Availability

Data will be made available on request.
