# Supplementary material for: Bio-inspired self-assembly of omega-3 fatty acids and peptides for responsive drug delivery
Source: Int J Pharm X. 2026 May 5;11:100558. doi: 10.1016/j.ijpx.2026.100558 (PMC13202583; doi:10.1016/j.ijpx.2026.100558)
Supplement: Supplementary file 1 — Supplementary material [file mmc1.docx]

Bio-inspired self-assembly of Omega-3 fatty acids and peptides for responsive drug delivery

Simone Braccia^1,2^, Luigi Alfano^3^, Maria Carmen Ragosta^4^, Rosa Bellavita^1^, Gabriella D’Auria, Emanuela Esposito^5^, Federica Donadio^5^, Sara Palladino^1^, Alessandro di Vaio ^3^Rosa Camerlingo^6^, Lucia Falcigno^1^, Annarita Falanga^7^, Michelino de Laurentiis,^3^ Antonio Giordano^2*^, Stefania Galdiero^1*^

¹Department of Pharmacy, School of Medicine, University of Naples Federico II, Naples, Italy

²Sbarro Institute for Cancer Research and Molecular Medicine, Center for Biotechnology, College of Science and Technology, Temple University, Philadelphia, PA, USA

³Department of Breast and Thoracic Oncology, Istituto Nazionale Tumori – IRCCS – Fondazione G. Pascale, Naples, Italy

⁴Scuola Superiore Meridionale (SSM), Clinical and Translational Oncology Program, University of Naples Federico II, Naples, Italy

⁵Institute of Applied Sciences and Intelligent Systems (ISASI), Naples Cryo-Electron Microscopy Laboratory – EYE LAB, National Research Council (CNR), Naples, Italy

⁶ Cell Biology and Biotherapy Unit, Istituto Nazionale Tumori-IRCCS-Fondazione G. Pascale, 80131 Naples, Italy

^7^Department of Agricultural Sciences, University of Naples Federico II, Portici, Italy

Email corresponding: stefania.galdiero@unina.it; antonio.giordano@temple.edu

**Table of contents**

**FIGURES (1-12)**

- **Figure S1** Schematic representation of peptide synthesis.
- **Figure S2-S8** HPLC chromatograms and ESI-MS of peptides.
- **Figure S9** NMR.
- **Figure S10** Calibration lines of Dox and PEM.
- **Figure S11** 3D confocal microscopy analysis of EPA-gH-Dox**.**
- **Figure S12** CD spectra of gH-EPA in buffer (in black) and in presence of 20% TFE (in red).
- **Figure S13** DLS analysis of SLNs EPA-gH-Dox.
- **Figure S14** DLS analysis of SLNs EPA-gH-TP-PEM stability after 72 h.
- **Figure S15** DLS analysis of SLNs EPA-gH-TP-PEM.

**TABLES (1-2)**

- **Table S1** Characterization of different formulations by DLS analysis using nanoemulsion-solvent evaporation.
- **Table S2** Characterization of different formulations by DLS analysis using the standard self-assembly procedure.





**Figure S1**. Schematic representation of peptide synthesis. (a) Fmoc deprotection with the solution of 30% piperidine in DMF, 2 x 10 min (b) Fmoc-amino acid (2 equiv) was added with *N,N′-*diisopropylcarbodiimide (DIC, 2 equiv) Oxyma pure (2 equiv) as coupling reagents, in DMF for 40 min, while the second coupling was performed with Fmoc-amino acid (2 equiv) HATU (2 equiv), DIPEA (4 equiv), in DMF for 40 min (c) MTT deprotection with the mixture 1 %  TFA and 5% TIS in  DCM. Peptide cleavage: TFA:TIS:H_2_O (95:2.5:2.5, v:v:v), 3h at rt. The protocol was used for the synthesis of all peptides.

**
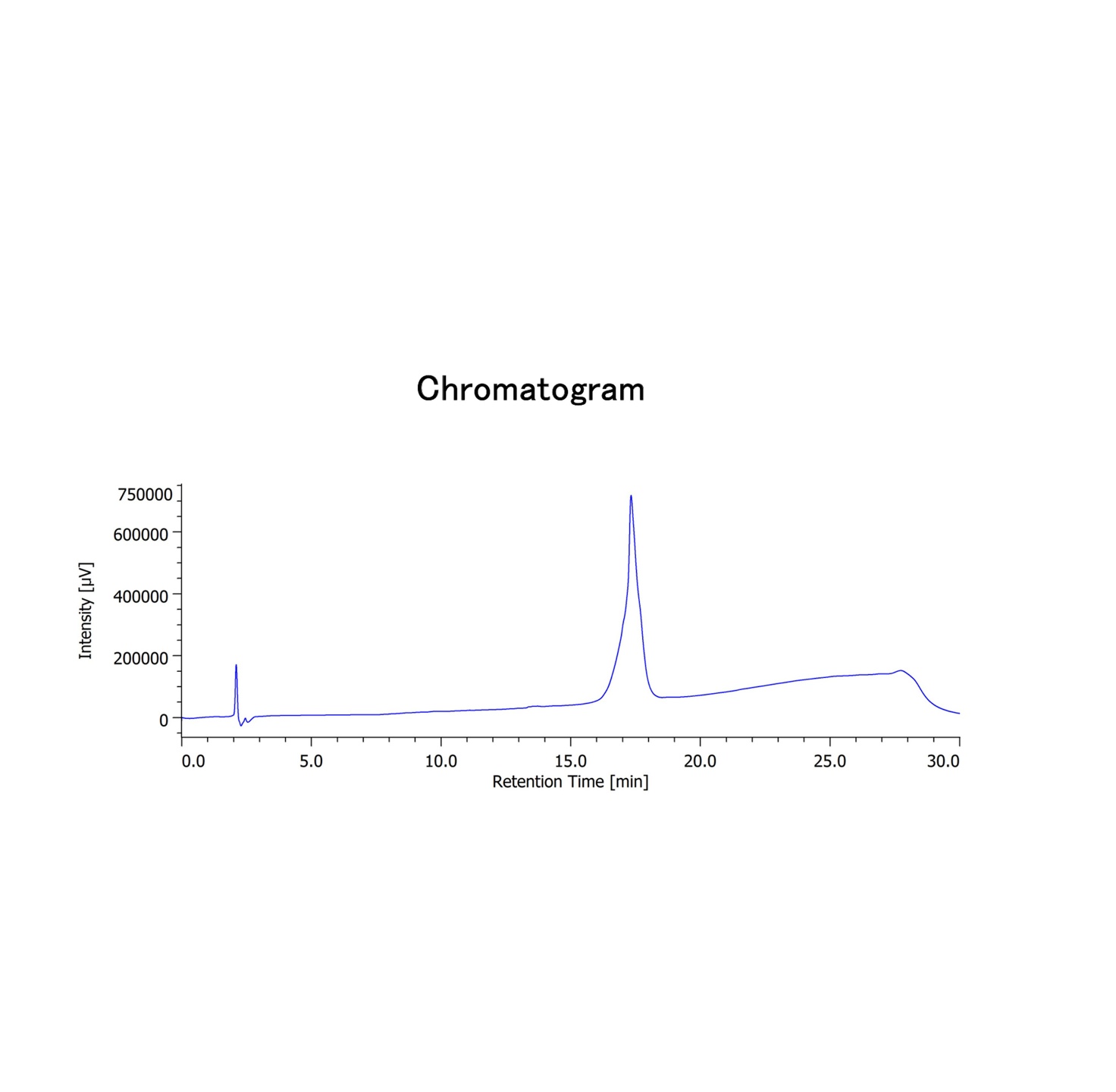
**

**Figure S2**. Chromatogram of peptide **gH-EPA**obtained by an analytical HPLC (Jasco LC-NetII/ADC) equipped with a Phenomenex Jupiter 4u Proteo 90A C18 column (150 mm × 4.6 mm, 5 μm, 100 Å), and monitored by UV detection at 220 nm. [linear gradient 10-90% MeCN (0.1% TFA) in H_2_O (0.1% TFA) over 15 min, flow rate of 1 mL/min]. Calculated mass: 2881.4. Found mass: [M+2H]+ /2=1441.3, [M+3H]+ /3=961.8, [M+4H]+ /4=721.7, [M+5H]+ /5=577.6.


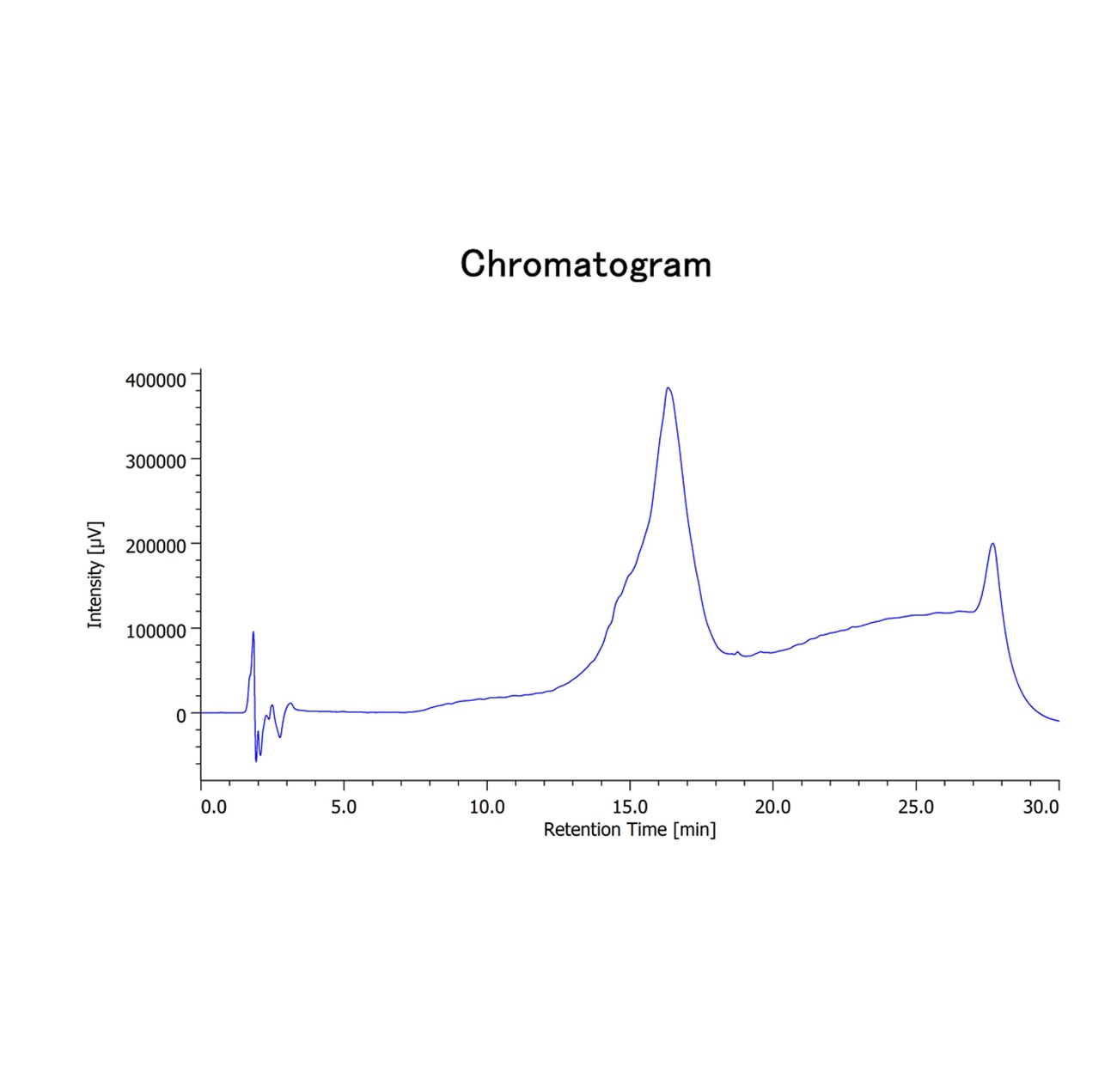


**Figure S3.** Chromatogram of peptide **Rho-gH-EPA**obtained by an analytical HPLC (Jasco LC-NetII/ADC) equipped with a Phenomenex Jupiter 4u Proteo 90A C18 column (150 mm × 4.6 mm, 5 μm, 100 Å), and monitored by UV detection at 220 nm. [linear gradient 10-90% MeCN (0.1% TFA) in H_2_O (0.1% TFA) over 15 min, flow rate of 1 mL/min]. Calculated mass: 3307.0. Found mass: [M+2H]+ /2=1654.1, [M+3H]+ /3=1103.3, [M+4H]+ /4=828.1, [M+5H]+ /5=662.5.


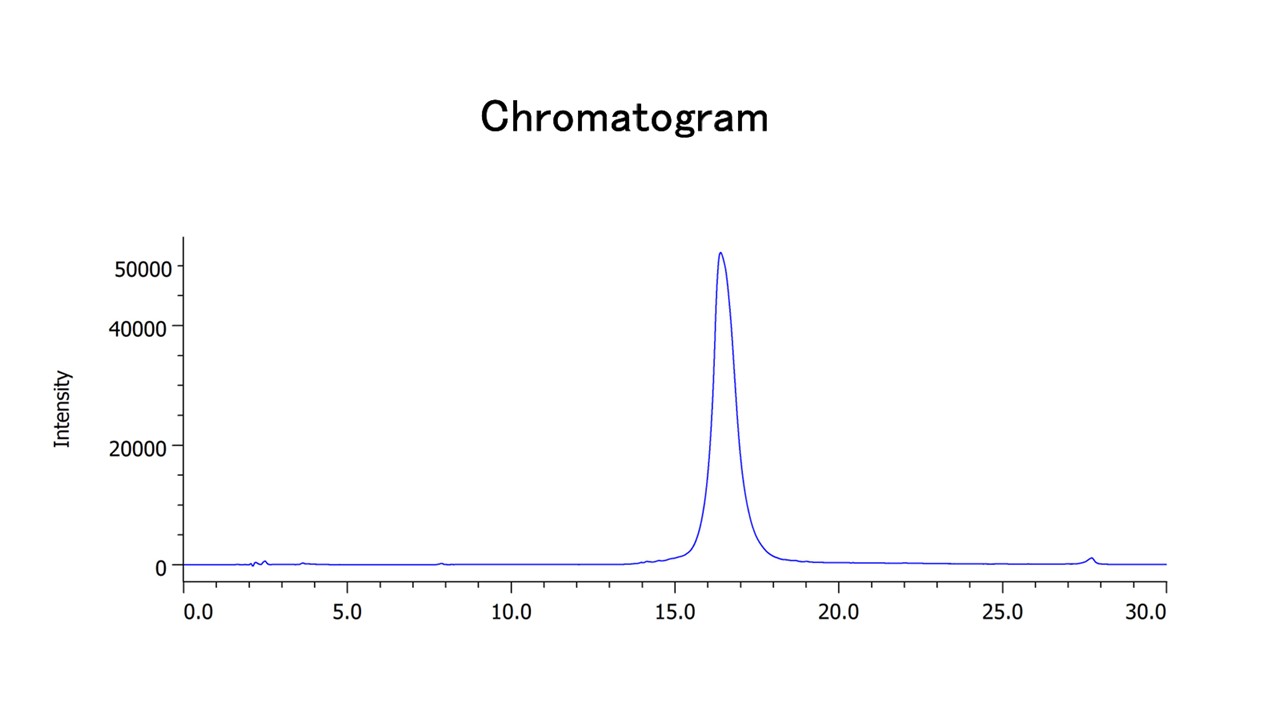


**Figure S4**. Chromatogram of peptide **Rho-gH-EPA**obtained by an analytical HPLC (Jasco LC-NetII/ADC) equipped with a Phenomenex Jupiter 4u Proteo 90A C18 column (150 mm × 4.6 mm, 5 μm, 100 Å), and monitored by UV detection at 576 nm [linear gradient 10-90% MeCN (0.1% TFA) in H_2_O (0.1% TFA) over 15 min, flow rate of 1 mL/min].


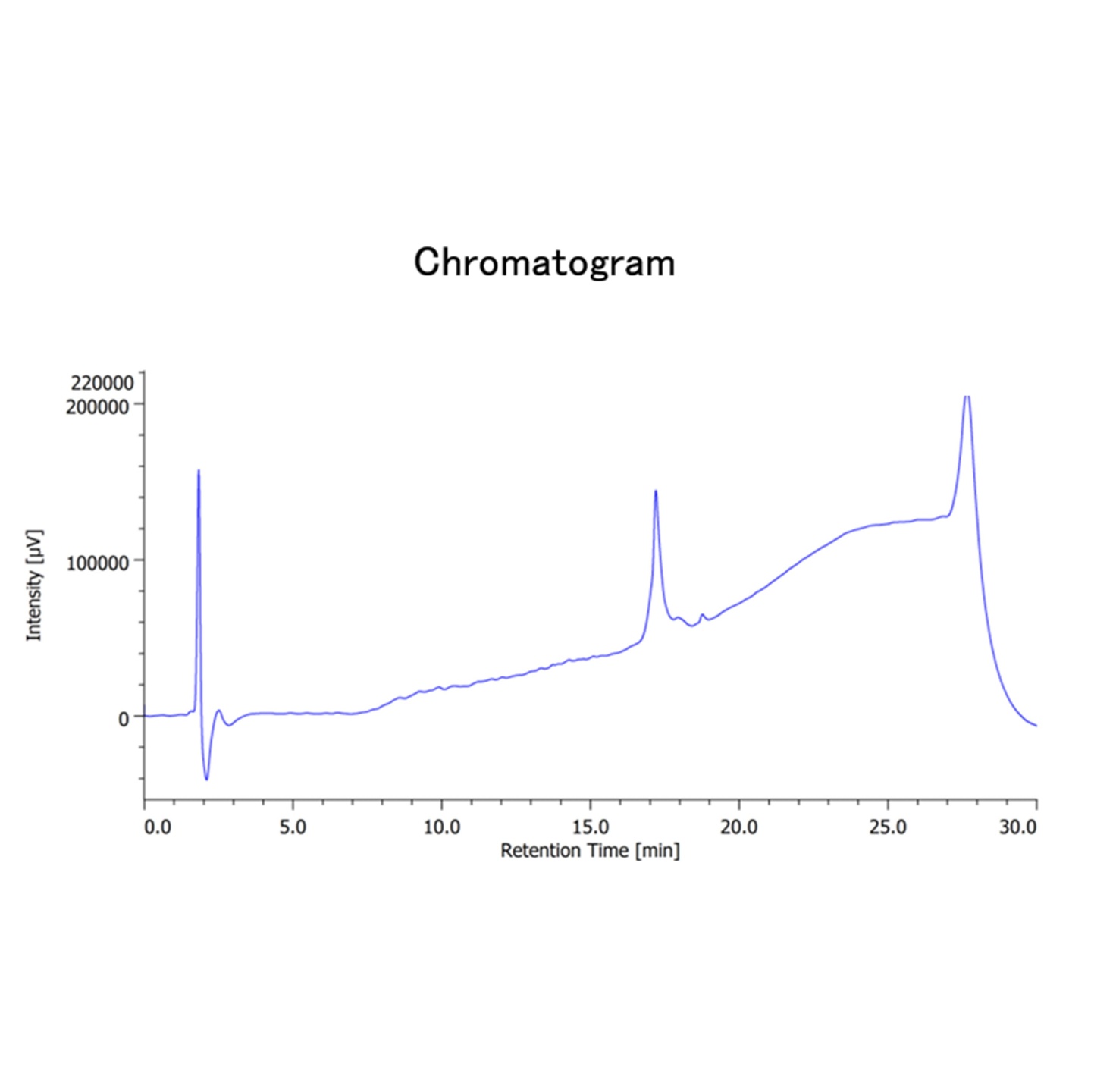


**Figure S5.** Chromatogram of peptide **falGea-EPA**obtained by an analytical HPLC (Jasco LC-NetII/ADC) equipped with Phenomenex Jupiter 4u Proteo 90A C18 column (150 mm × 4.6 mm, 5 μm, 100 Å), and monitored by UV detection at 220 nm. [linear gradient 10-90% MeCN (0.1% TFA) in H_2_O (0.1% TFA) over 15 min, flow rate of 1 mL/min]. Calculated mass: 1189.5. Found mass: [M+1H]+ /1=1189.8, [M+2H]+ /2=595.7,. [M+3H]+ /3=961.8, [M+4H]+ /4=721.7.


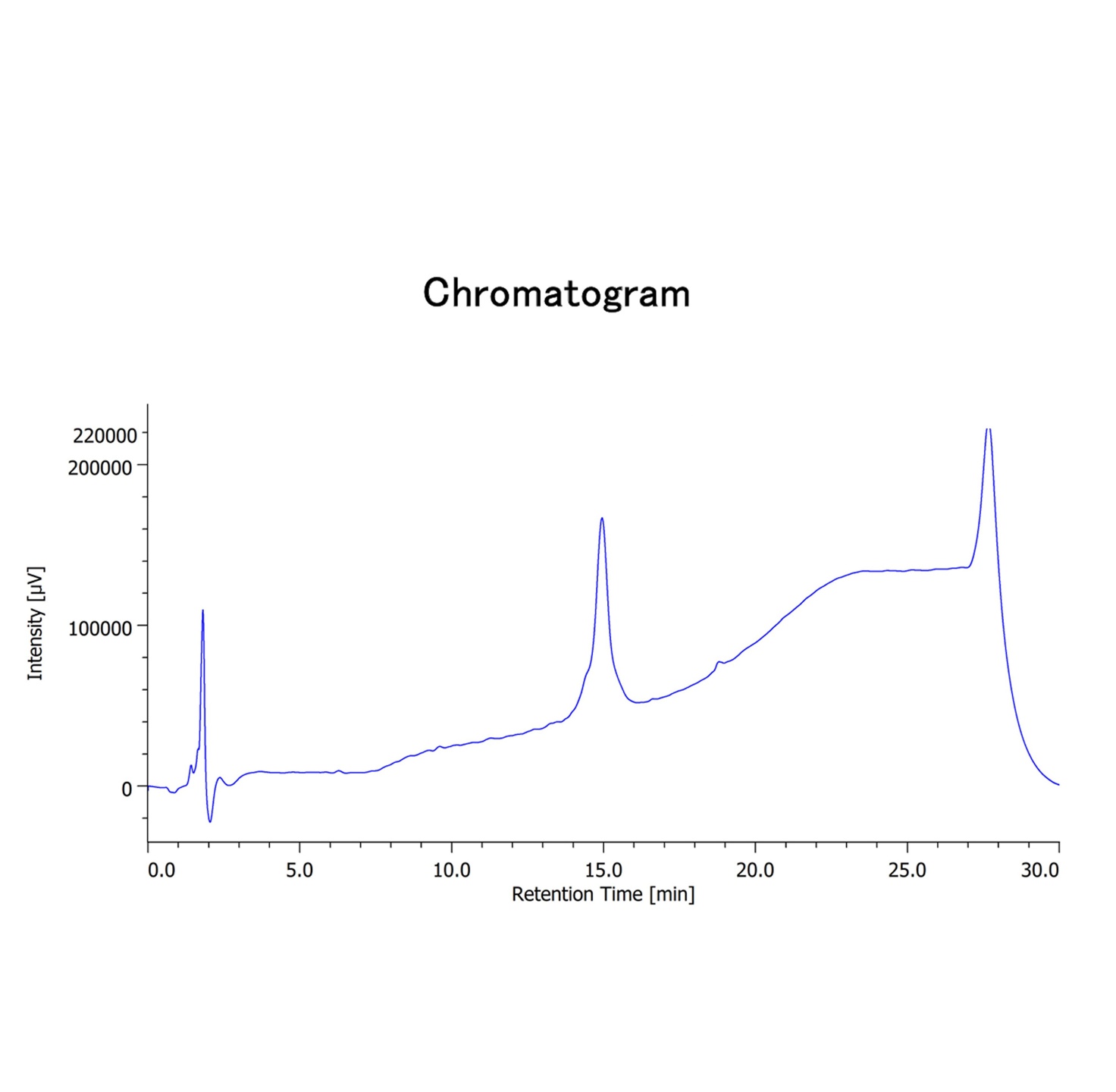


**Figure S6.** Chromatogram of peptide **Dox-EPA-NC**obtained by an analytical HPLC (Jasco LC-NetII/ADC) equipped with a Phenomenex Jupiter 4u Proteo 90A C18 column (150 mm × 4.6 mm, 5 μm, 100 Å), and monitored by UV detection at 220 nm. [linear gradient 10-90% MeCN (0.1% TFA) in H_2_O (0.1% TFA) over 15 min, flow rate of 1 mL/min]. Identity confirmed with NMR (Figure S8 Panel A).

**
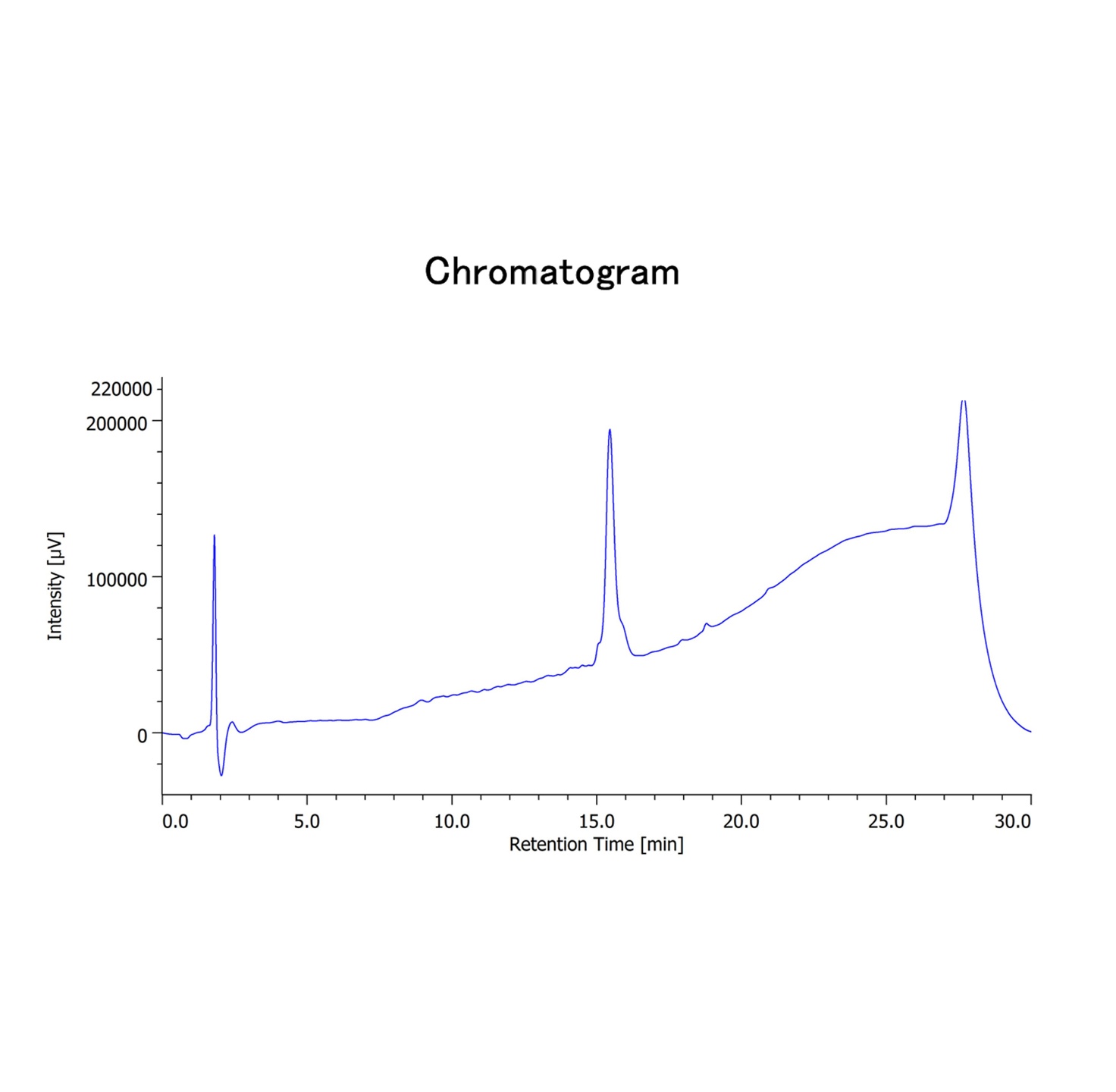
**

**Figure S7.** Chromatogram of peptide **Dox-EPA** obtained by an analytical HPLC (Jasco LC-NetII/ADC) equipped with a Phenomenex Jupiter 4u Proteo 90A C18 column (150 mm × 4.6 mm, 5 μm, 100 Å), and monitored by UV detection at 220 nm. [linear gradient 10-90% MeCN (0.1% TFA) in H_2_O (0.1% TFA) over 15 min, flow rate of 1 mL/min]. Identity confirmed with NMR (not shown).


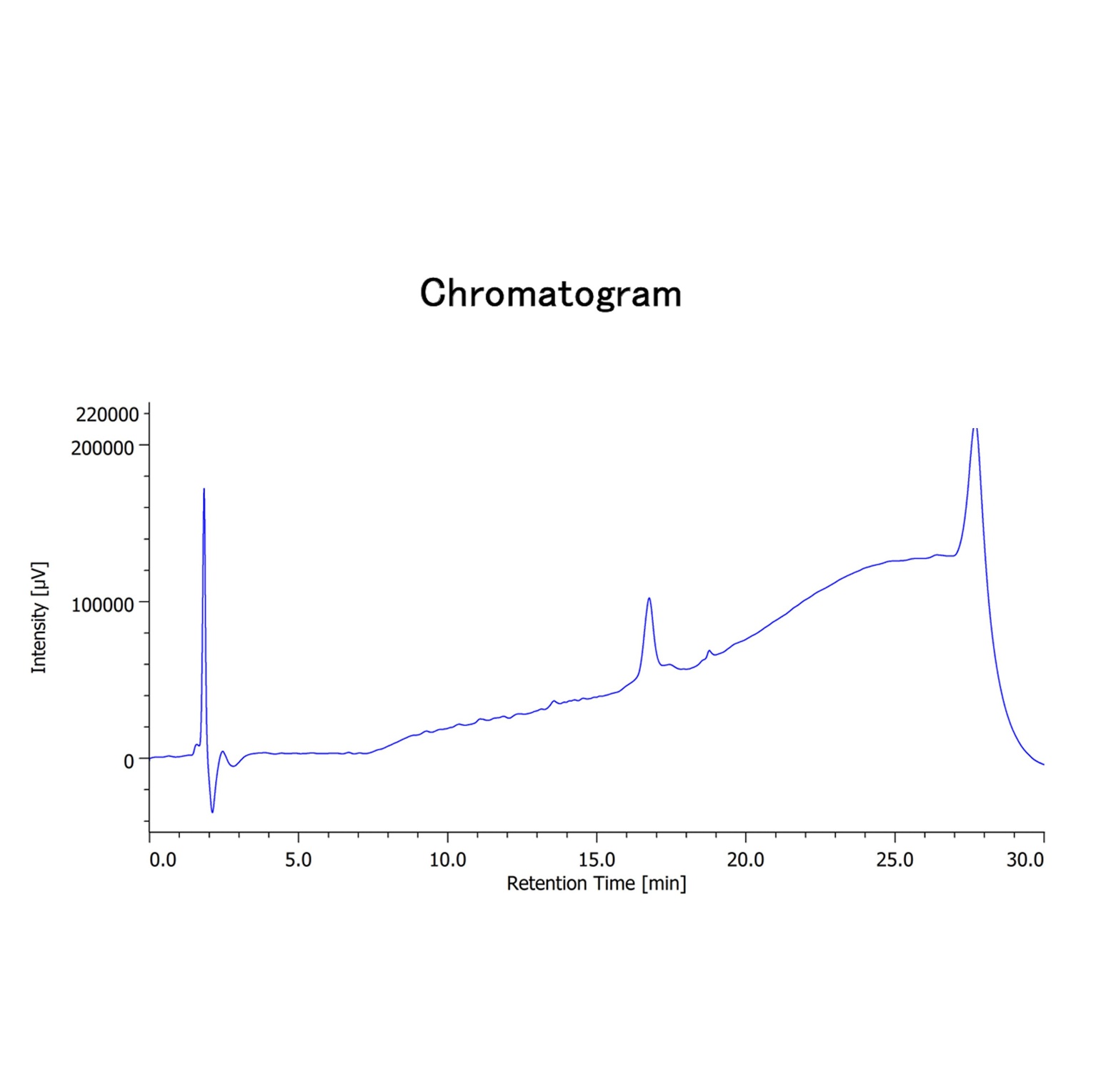


**Figure S8.** Chromatogram of peptide **PEM-EPA** obtained by an analytical HPLC (Jasco LC-NetII/ADC) equipped with a Phenomenex Jupiter 4u Proteo 90A C18 column (150 mm × 4.6 mm, 5 μm, 100 Å), and monitored by UV detection at 220 nm. [linear gradient 10-90% MeCN (0.1% TFA) in H_2_O (0.1% TFA) over 15 min, flow rate of 1 mL/min]. Identity confirmed with NMR (Figure S9 Panel B).


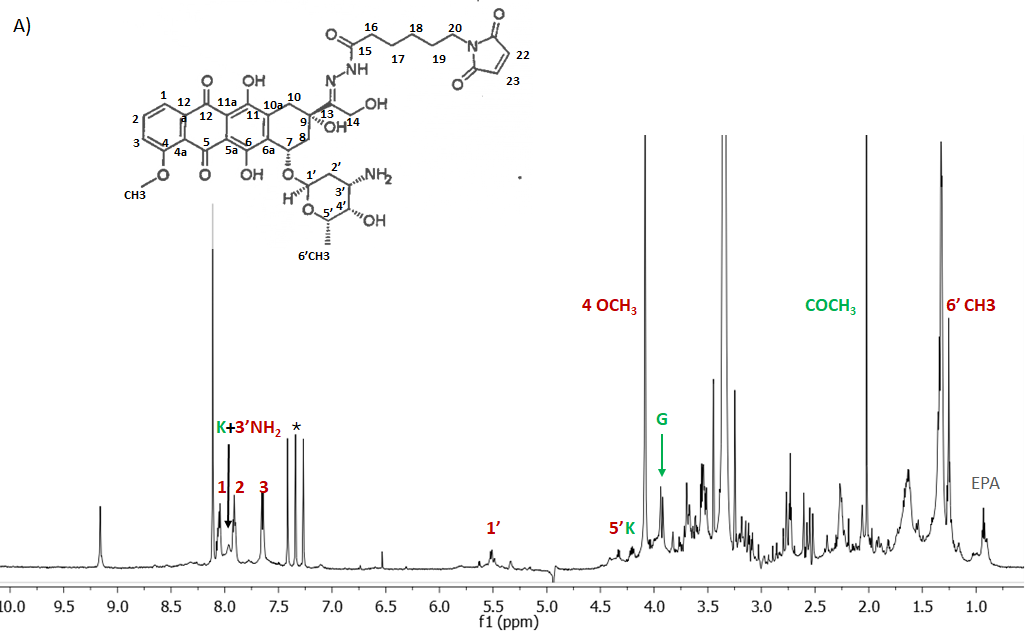


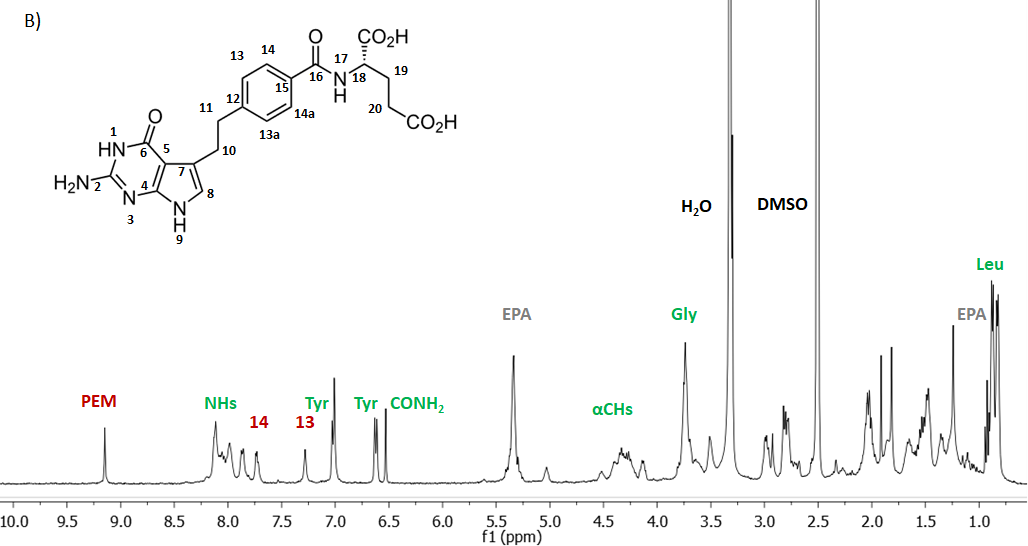


**Figure S9.** NMR spectra in CD_3_OH at 298 K of Dox-EPA-NC (Panel A) and PEM-EPA (Panel B). Schematic representations of Dox and PEM structures are inserted into Panel A and B, respectively. Some resonances belonging to peptide (green), PEM or Dox (red) and EPA (dark grey) are indicated.


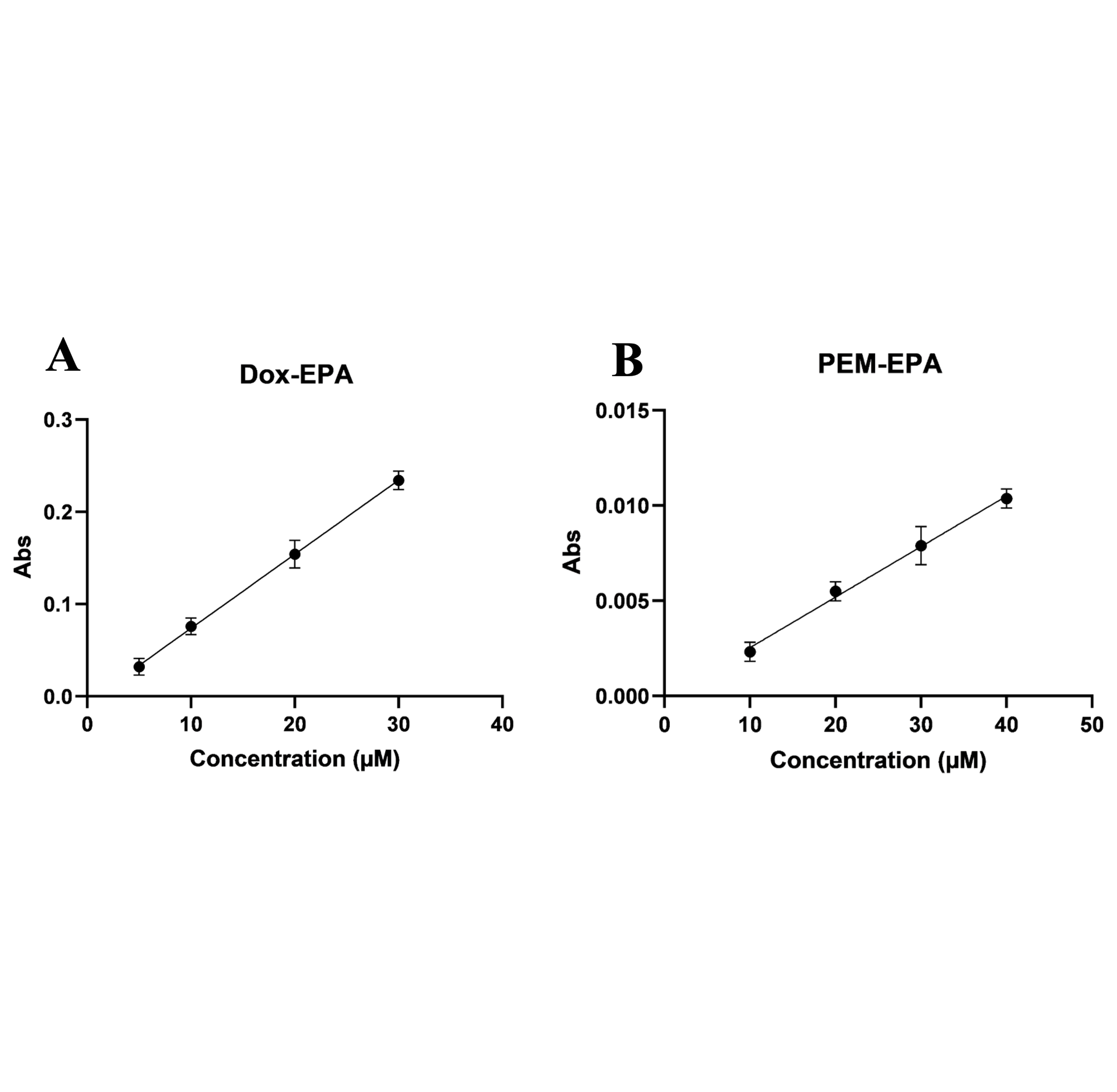


**Figure S10.** Dox-EPA was quantified by UV-Vis spectroscopy using a Jasco V-750 spectrophotometer. To plot the calibration curve, solutions of the Dox-EPA peptide were prepared at increasing concentrations, and UV-Vis spectra were recorded in the range between 400 and 600 nm; the values used to plot the curve were the absorbance values at 480 nm (Panel A).  PEM-EPA was quantified by UV-Vis spectroscopy using a Jasco V-750 spectrophotometer. To plot the calibration curve, solutions of the PEM-EPA peptide were prepared at increasing concentrations, and UV-Vis spectra were recorded in the range between 225 and 300 nm; the values used to plot the curve were the absorbance values at 275 nm (Panel B).

**Figure S11.** 3D confocal microscopy analysis revealing the intracellular aggregation of EPA-gH-Dox at Dox concentration higher than 6%. Doxorubicin fluorescence is shown in orange, and nuclei are stained in blue with Hoechst.


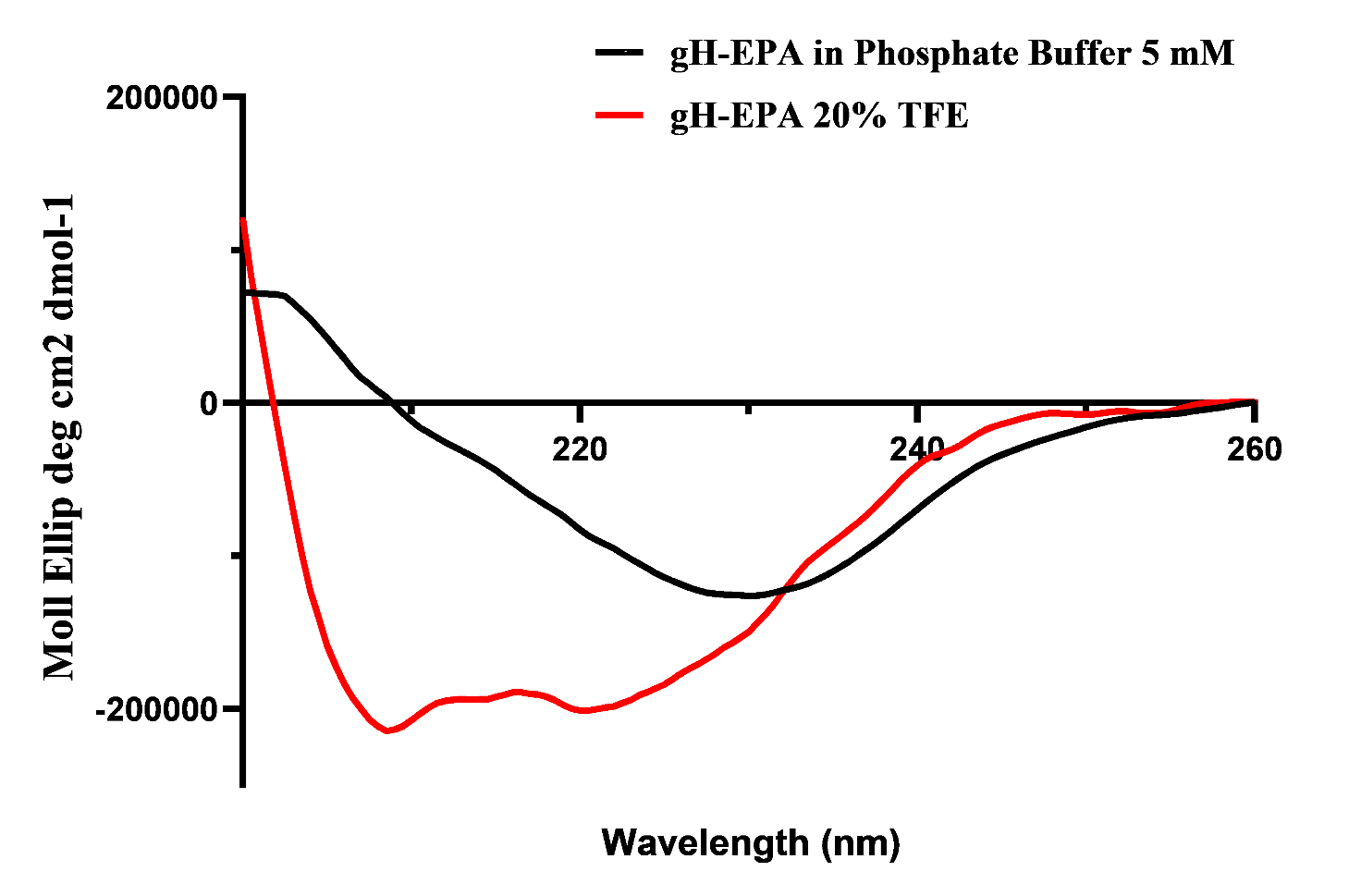


**Figure S12.** CD spectra of gH-EPA in buffer (in black) and in presence of 20% TFE (in red).

*
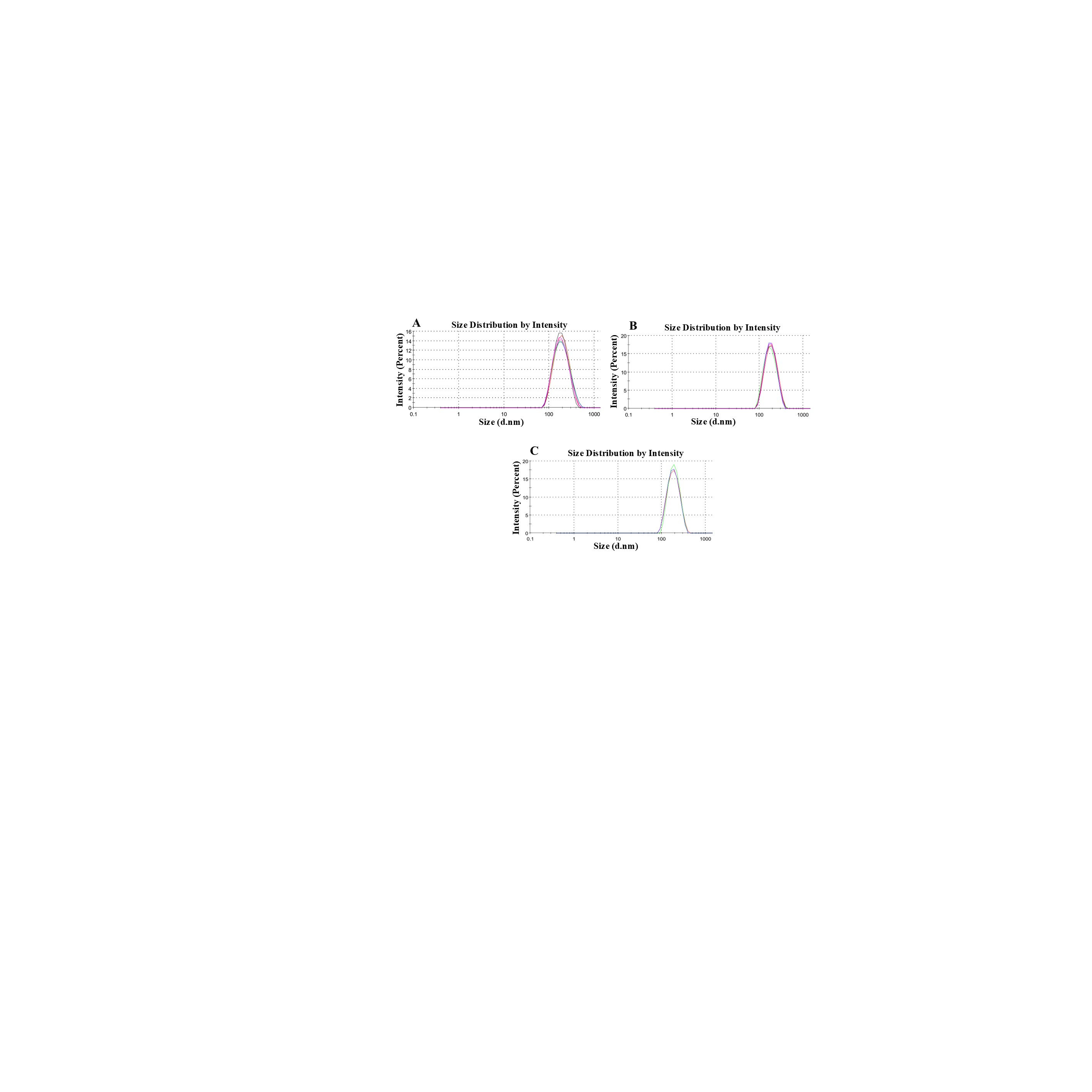
*

**Figure S13.** DLS analysis of SLNs EPA-gH-Dox, effects of dilution from 350 μM to 11 μM (Panel A), ionic strength from 0 mM NaCl to 5 mM NaCl (Panel B), pH 3, pH 7, pH 10 (Panel C).

**
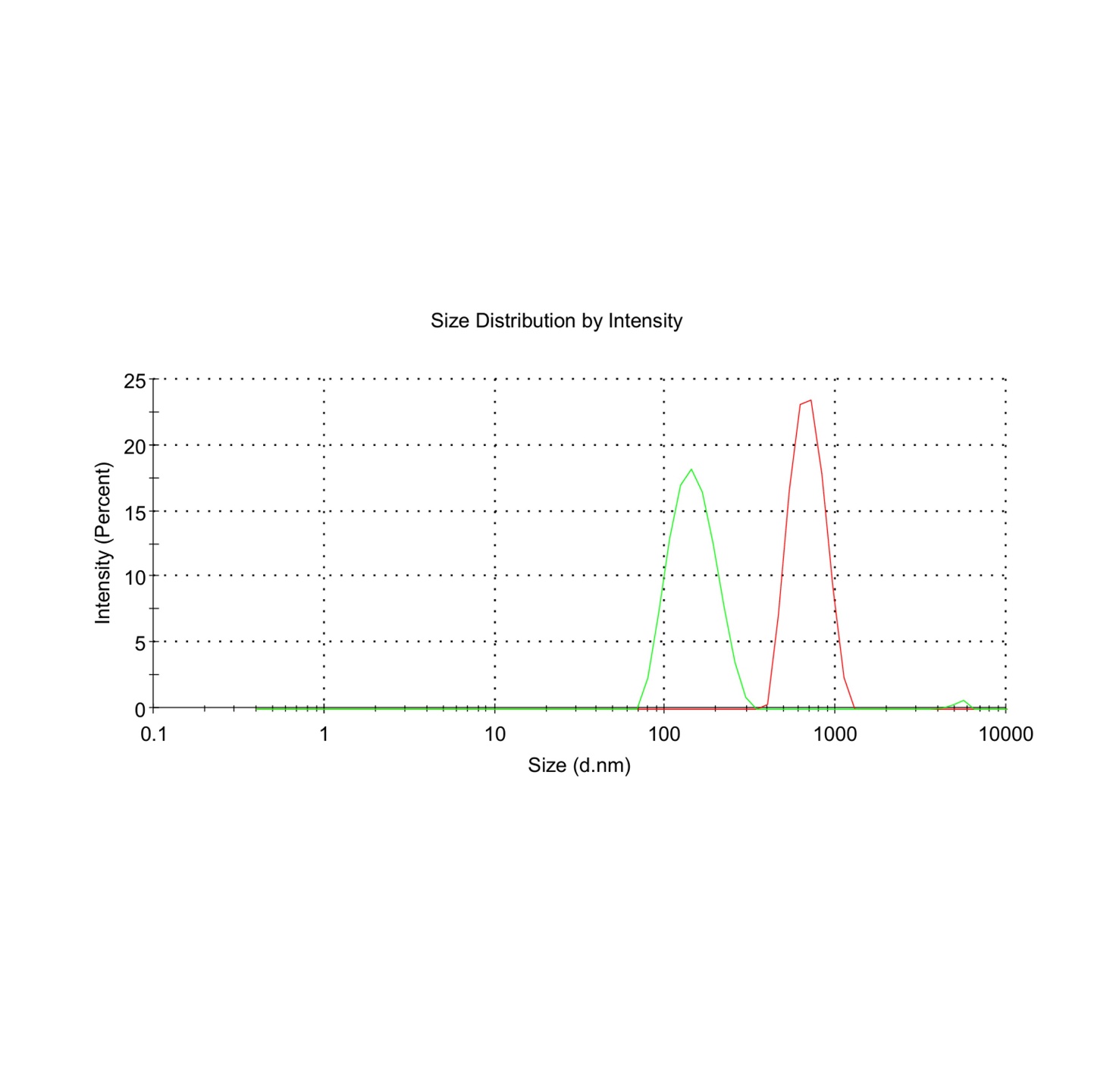
**

**Figure S14.** DLS analysis of EPA-gH-TP-PEM SLNs at time 0 (green) and after 72 hours of storage (red), assessing nanoparticle stability.


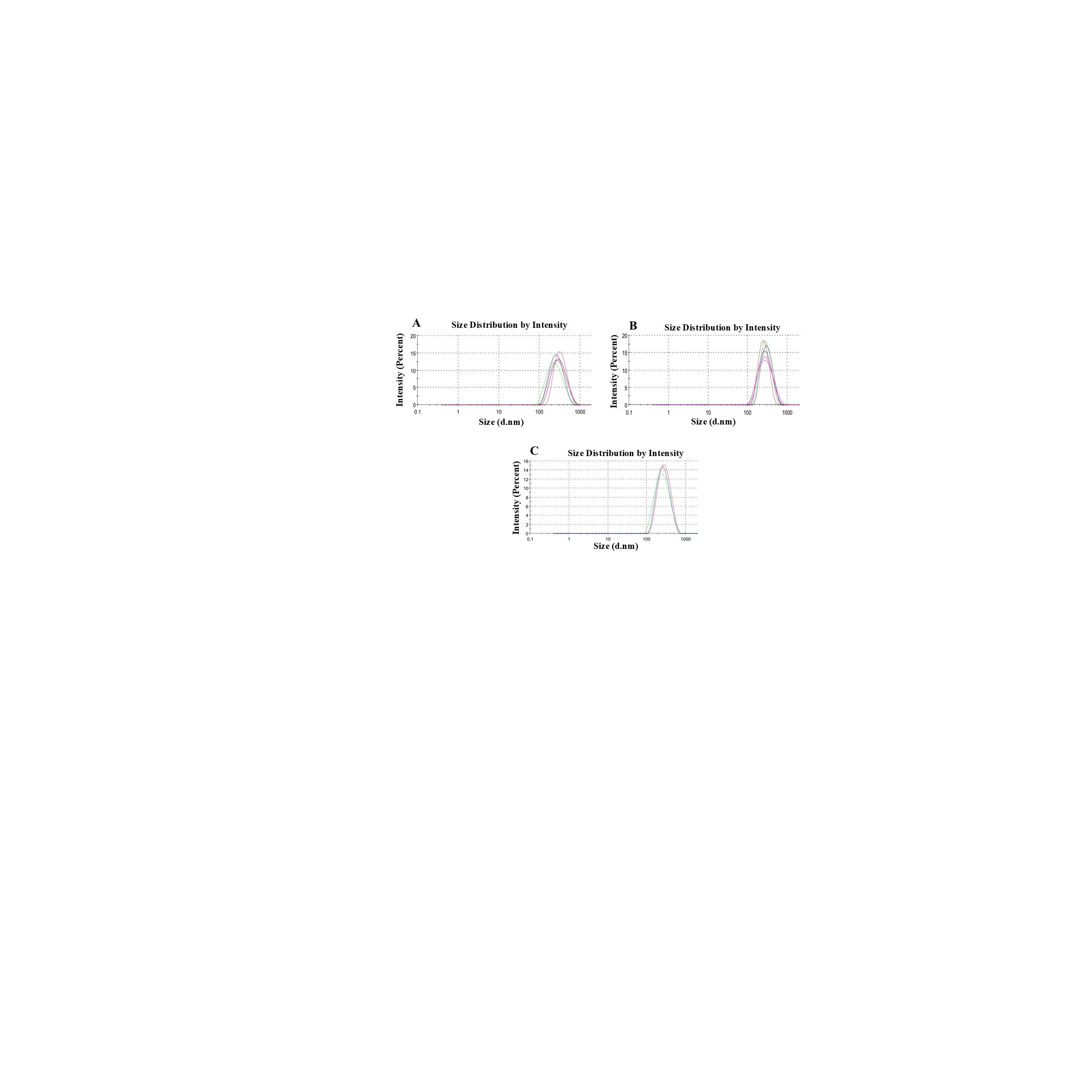


**Figure S15.** DLS analysis of SLNs EPA-gH-TP-PEM, effects of dilution from 250 μM to 12.5 μM (Panel A), ionic strength from 0 mM NaCl to 5 mM NaCl (Panel B), pH 3, pH 7, pH 10 (Panel C).

**Table S1** Characterization of different formulations by DLS analysis using nanoemulsion-solvent evaporation.

| Formulation | Lipid/Peptide | Concentration (μM) | Size (d. nm) | PdI |
| --- | --- | --- | --- | --- |
| EPA | - | 10 | 185±1 | 0.14± 0.01 |
| EPA | - | 100 | 163±5 | 0.34±0.01 |
| EPA | - | 350 | 173±10 | 0.19±0.04 |
| EPA | - | 5000 | 194±12 | 0.06±0.01 |
| EPA (after 7 days) | - | 5000 | 193±1 | 0.10±0.05 |
| EPA (after 30 days) | - | 5000 | 126± 6 | 0.30±0.02 |
| EPA: gH-EPA | 9:1 | 10 | 390± 2 | 0.25±0.01 |
| EPA: gH-EPA | 9:1 | 100 | 419± 2 | 0.44±0.01 |
| EPA: gH-EPA | 9:1 | 350 | 350± 1 | 0.23±0.04 |
| EPA: gH-EPA | 9.7:0.3 | 10 | 130± 2 | 0.30±0.01 |
| EPA: gH-EPA | 9.7:0.3 | 100 | 137±3 | 0.34±0.01 |
| EPA: gH-EPA | 9.7:0.3 | 350 | 193±5 | 0.26±0.02 |

**Table S2** Characterization of different formulations by DLS analysis using the standard self-assembly procedure.

| Formulation | Concentration (μM) | Size (d. nm) | PdI |
| --- | --- | --- | --- |
| EPA | 10 | 417±1 | 0.25±0.01 |
| EPA | 15 | 435±2 | 0.32±0.01 |
| EPA | 20 | 629±30 | 0.43±0.01 |
